# Analysis of Results of Specific IgE in 100 Atopic Dermatitis Patients with the Use of Multiplex Examination ALEX2—Allergy Explorer

**DOI:** 10.3390/ijms22105286

**Published:** 2021-05-17

**Authors:** Jarmila Čelakovská, Josef Bukač, Eva Cermákova, Radka Vaňková, Hana Skalská, Jan Krejsek, Ctirad Andrýs

**Affiliations:** 1 Department of Dermatology and Venereology, Faculty Hospital and Medical Faculty of Charles University, 50002 Hradec Králové, Czech Republic; 2 Department of Medical Biophysic, Medical Faculty of Charles University, 50002 Hradec Králové, Czech Republic; bukacjosef@seznam.cz (J.B.); CermakovaE@lfhk.cuni.cz (E.C.); 3 Department of Clinical Immunology and Allergy, Faculty Hospital and Medical Faculty of Charles University, 50002 Hradec Králové, Czech Republic; vankovr@lfhk.cuni.cz (R.V.); jan.krejsek@fnhk.cz (J.K.); ctirad.andrys@fnhk.cz (C.A.); 4 Department of Informatics and Quantitative Methods, Faculty of Informatics and Management, University of Hradec Kralove, 50003 Hradec Králové, Czech Republic; hana.skalska@uhk.cz

**Keywords:** atopic dermatitis, ALEX2 Allergy Explorer, molecular components, severity of atopic dermatitis, disturbed epidermal barrier, asthma bronchiale, allergic rhinitis

## Abstract

Background and aim: Progress in laboratory diagnostics of IgE-mediated allergy is the use of component-resolved diagnosis. Our study analyses the results of specific IgE to 295 allergen reagents (117 allergenic extracts and 178 molecular components) in patients suffering from atopic dermatitis (AD) with the use of ALEX2 Allergy Explorer. Method: The complete dermatological and allergological examination, including the examination of the sensitization to molecular components with ALEX2 Allergy Explorer testing, was performed. The statistical analysis of results was performed with these methods: TURF (total unduplicated reach and frequency), best reach and frequency by group size, two-sided tests, Fisher’s exact test, and chi-square test (at an expected minimum frequency of at least 5). Results: Altogether, 100 atopic dermatitis patients were examined: 48 men, 52 women, the average age 40.9 years, min. age 14 years, max. age 67 years. The high and very high level of specific IgE was reached in 75.0% of patients to 18 molecular components: from PR-10 proteins (Aln g 1, Bet v 1, Cor a1.0103, Cor a1.0401, Fag s 1), lipocalin (Can f 1), NPC2 family (Der f 2, Der p 2), uteroglobin (Fel d 1), from Alternaria alternata (Alt a 1), Beta expansin (Lol p 1, Phl p 1), molecular components from Timothy, cultivated rye (Secc pollen) and peritrophin-like protein domain Der p 23. The high and very high level of specific IgE to other lipocalins (Fel d 7, Can f 4), to arginine kinase (Bla g 9, German cockroach), and to allergen extracts Art v (mugwort), and Cyn d (Bermuda grass) reached 52.0% of patients. The severity of AD is in significant relation to the sensitization to molecular components of storage mites (Gly d 2, Lep d 2—NPC2 family), lipocalins (Can f 1, Can f 2, Can f 4, and Can f 6), arginine kinase (Asp f 6, Bla g 9, Der p 20, Pen m 2), uteroglobin (Fel d 1, Ory c 3), Mn superoxide dismutase (Mala s 11), PR-10 proteins (Fag s 1, Mal d 1, Cor a 1.0401, Cor a 1.0103), molecular components of the peritrophin-like domain (Der p 21, Der p 23), and to Secc pollen. In the subgroup of patients suffering from bronchial asthma, the significant role play molecular components from house dust mites and storage mites (Lep d 2, Der p 2, Der f 2—NPC2 family), cysteine protease (Der p 1), peritrophin-like protein domain (Der p 21, Der p 23), enolase from Alternaria alternata (Alt a 6), and Beta expansin Phl p 1. Conclusion: The results of our study demonstrate the detailed profile of sensitization to allergens reagents (allergen extract and molecular components) in patients with atopic dermatitis. We show the significance of disturbed epidermal barrier, resulting in increased penetration of allergens. We confirmed the significant relationship between the severity of AD, the occurrence of bronchial asthma and allergic rhinitis, and high levels of specific IgE to allergen reagents. Our results may be important for regime measures and immunotherapy; Der p 23 shall be considered as an essential component for the diagnosis and specific immunotherapy of house dust mite allergy.

## 1. Introduction

Atopic dermatitis is a common inflammatory skin disease affecting up to 25% of children and up to 10% of adults in Western industrialized countries. Skin barrier abnormalities have been suggested to play an essential role in the initiation of early atopic dermatitis (AD). Antigen penetration through a compromised barrier likely leads to increased innate immune responses, antigen-presenting cell stimulation, and priming of overt cutaneous disease. In a T_H_2-promoting environment, T-cell/B-cell interactions occurring in regional lymph nodes lead to excessive IgE switches. Concurrent redistribution of memory T cells into the circulation not only leads to exacerbation of AD through T-cell skin infiltration but also spreads beyond the skin to initiate the atopic march, which includes food allergy, bronchial asthma, and allergic rhinitis [1,2]. The role of filaggrin (FLG), a protein contained in the granular layer of the epidermis regulating the aggregation of keratin filaments, was evidenced in atopic dermatitis as several loss-of-function mutations in *FLG* gene or FLG deficiency contribute to epidermal barrier dysfunction and was strongly associated with AD [1,2]. Mechanisms for developing atopic comorbidities after AD onset are poorly understood but can involve the impaired cutaneous barrier, which facilitates cutaneous sensitization. The association can also be driven or amplified in susceptible subjects by a systemic T_H_2-dominant immune response to cutaneous inflammation. However, these associations might merely involve shared genetic loci and environmental triggers, including microbiome dysregulation, with the temporal sequence reflecting tissue-specific peak time of occurrence of each disease, suggesting more clustering of disorders than a march [1,2,3,4]. In genetically susceptible individuals, the immune response to allergens is based mainly on specific IgE and type 2 cytokines, such as IL-4, IL-5, and IL-13. The specific IgE primary response to allergens is exclusively developed through the adaptive pathways of immunity. After sensitization, the re-exposure to allergens initiates the IgE-dependent inflammatory cascade, which involves additional cells and cytokines that actively participate in the inflammatory reaction [5,6,7,8]. Several factors, sometimes acting concomitantly, modulate the IgE primary response and sensitization process, including the levels of allergen exposure (which in turn depends on protein production by the source, permanence in the house dust and the air, effects of other enzymes upon the allergen), the persistence of exposure, age of exposed individuals, boosting of the IgE responses by conditions such as air pollution or helminth infections, and stimulation of Th1 responses by bacterial and other products [5,6,7,8,9]. The capacity of proteins to became allergens is unknown. Pro-inflammatory properties are not clearly stated. There is no common characteristic of being an allergen [9]. The broadest definition of an allergen is that it is any molecule that binds IgE antibodies [9]. Allergens can differ in several ways; most, but not all, allergens are sensitizing, which is defined as the ability to induce allergen-specific IgE antibodies. Nonsensitizing allergens can only cause allergic symptoms if previous contact with a related (cross-reactive) allergen has caused sensitization. A prototypic example of a sensitizer is birch allergen Bet v 1, and a cross-reactive non-sensitizer is the homologous apple allergen Mal d 1 [9]. Allergens have diverse pro-inflammatory properties that can increase their allergenic activity; several innate non-IgE-mediated inflammatory mechanisms have been reported, including the ability to bind adjuvants or to stimulate the innate immunity via toll-like receptors and other receptors in the bronchial epithelium. Der p 1 and Der p 2 are suitable examples of molecules with high allergenic activity that could be due in part to their ability to stimulate both innate and adaptive pathways of the allergic response [10,11,12,13,14,15,16].

Currently, the diagnostic procedure of type I allergy includes the evaluation of patients’ symptoms, followed by screening skin prick tests with a panel of respiratory allergens and/or food allergens and conclusive specific IgE testing in the serum of the patients. In this setting, on the one hand, relevant allergens could be missed. On the other hand, sometimes skin testing is not possible due to inflamed or atopic skin, a matter that is hotly debated. Molecular allergy testing, particularly using allergen microarrays, is currently being implemented into daily diagnostic work-up of allergies. Molecular allergy diagnosis using singleplex allergens or multiplex allergen microarrays are typical methods of precision medicine [9,17,18,19]. They enhance the specificity of IgE diagnosis in polysensitized respiratory allergies, can be applied in food allergies and atopic dermatitis, and may even reveal unexplained anaphylaxis [9]. The molecular components are recombinant proteins with different IgE binding capacities [9,17,18,19]. ALEX2 Allergy Explorer is an in vitro assay for the measurement of allergen-specific IgE antibodies in human plasma [18,19]. It is intended to aid in the diagnosis of IgE-mediated allergic disorders [18,19]. The major advantage of ALEX2 is the comprehensive IgE pattern obtained with a minute amount of serum [18,19]. The macroarray nanotechnology-based immunoassay used as a molecular allergy explorer (ALEX^®^; MacroArray Diagnostics, Wien, Austria) is the latest launched in vitro multiplex tool for precision medicine in allergy diagnosis. It is based on a state-of-the-art proprietary nano-bead technology. This new array contains 295 allergen reagents (117 allergenic extracts and 178 molecular components), with a large majority of aeroallergen families and cross-reactive food allergens being represented. This in vitro allergy explorer is the first in vitro multiplex allergy test allowing simultaneous measurement of total IgE and specific IgE against a plethora of allergen extracts and molecular allergens. The combination of second- and third-level assays in the same immunoassay allows to define the presence of IgE sensitization, whether it is genuine or cross-reactive, and saves time and costs, particularly in polysensitized patients and/or with pollen food syndromes [19]. The ALEX^®^ in vitro allergy test core technology is based on a two-phased manufacturing process and it represents a multiplex ELISA-based test with proven immunoassay chemistry and detection methods [19].

Depending on the geographic and climatic region and vegetation, allergenic plants are characteristic for certain areas, and the pollen concentrations of various plant species depend on the fenophase of each single species and, most of all, on the climatic and meteorological conditions of a certain area. It is precisely because of these specific characteristics that the hypersensitivity of patients to various types of pollen allergens differs according to the geographic regions [20].

IgE results, whether to whole extracts or components, can be classified as primary or caused by cross-sensitization. Some allergens are predominantly primary, whereas others are predominantly encountered as pan-allergens that cause cross-sensitization. IgE sensitization is not synonymous with allergy, and significantly elevated IgE levels may be encountered in people who do not have a clinical allergy. It is thus imperative that the interpretation of laboratory results to multiple allergens endeavors to distinguish between clinically irrelevant cross-sensitization and clinically relevant cross-reactivity [20].

In our previous studies, we evaluated in patients suffering from AD the occurrence of sensitization to molecular components with the use of ISAC multiplex testing. ImmunoCAP ISAC is a solid-phase multiple immunoassay, which enables the determination of 112 different components from 51 allergen sources. According to our results, sensitization to molecular components of NPC2 proteins family, uteroglobin, lipocalin, aspergillus, and sensitization to molecular components of Timothy was recorded with the significantly higher occurrence in a severe form of AD [21,22].

## 2. Results

A total of 100 atopic dermatitis patients were included in the study (48 men and 52 women with the average age 40.9 years: min. age 14 years, max. age 67 years, and with the average SCORAD 39, s.d.13.1 points). The mild form of AD was recorded in 14 patients (14.0%), moderate form in 61 patients (61.0%), severe form in 25 patients (25.0%); 55 patients (55.0%) suffer from bronchial asthma and 74 patients (74.0%) suffer from allergic rhinitis. Positive family history about atopy was recorded in 48 patients (48.0%). The onset of AD before 5 years of age in 61 patients (61.0%) and the persistent eczematic lesions in 57 patients (57.0%). The characteristics of patients are shown in Table 1.

We evaluated the sensitization rate of positive results of specific IgE to 295 allergen reagents (117 allergenic extracts and 178 molecular components) in the whole group of patients (Table 2). The highest number of positive results of specific IgE (Classes 1, 2, 3, 4) rached 57% of patients) to grass-species specific component Phl p 1 (Timothy, Beta expansin). High sensitization to other molecular components and extracts were recorded: in 49% of patients to Fag s 1 (European beech), in 48% to Cor a 1.0103, Cyn d (Bermuda grass, Beta expansin), Cor a pollen (hazel pollen), in 45% to Der f 2 (house dust mite, NPC2 family), Fra a 1 + 3 (PR-10 protein, strawberry), Phl p 2 (grass group II, Timothy), in 44% to Cor a 1.0401 (hazelnut, PR-10 protein), Der p 2 (house dust mite, NPC2 family), Fel d 1 (Cat, uteroglobin), in 43% to Aln g 1 (alder, PR-10 protein), in 42% to Pas n (bahia grass), Phl p 5.0101, Phl p 6 (Timothy), and in 41% to Lep d (storage mite, NPC2 family 2), Mal d 1 (apple, PR-10 protein), (Table 2).

We also show the results of specific IgE to 295 allergen reagents (117 allergenic extracts and 178 molecular components) according to the Classes 0, 1, 2, 3, 4 (Table S1) 100 atopic dermatitis patients (=100%), No sensitization was confirmed to Ani s1 (anisakis simplex, unknown function), Bos d 5 (cow’s milk, beta-lactoglobulin), Cup s (cypress), Fag e 2 (common buckwheat, 2S albumin), Gly m 8 (soybean, 2S albumin), Mor r (mulberry), Ori v (oregano), Ovi a epithelia (sheep epithel), Ovi a meat (sheep meat), Raj c (thornback ray), Sin (mustard), Tri a 19 (omega-5-gliadin), Tri s (spelt), Table 2 and Table S1). Allergen extracts and molecular components with the positive results of specific IgE in Class 1 (recorded in more than 10% of patients): Hel a (sunflower seed), Acam (acacia), Act d 2 (kiwi, thaumatin-like protein), Api g 2 (celery, non-specific lipid transfer protein), Api m 1 (phospholipase A2, honey bee venom), Phr c (reed), Pla l (English plantain), Bla g 4 (lipocalin, German cockroach), Cla h 8 (Mannitol dehydrogenase, Cladosporium herbarum), Cyn d (Bermuda grass), Ves v 5 (antigen 5, yellow jacket venom). Allergen extracts and molecular components with the positive results of specific IgE in Class 2 (recorded in more than 10% of patients): Ara h 8 (peanuts, PR-10 protein), Cor a 1.0401 (PR-10 protein, hazel pollen), Cyn d 1 (Beta expansin, Bermuda grass), Der p 7 (mites group 7, bactericidal permeability-increasing-like protein, house dust mite), Pas n (Bahia grass), Secc pollen (cultivated rye, pollen), Fel d 7, Fra a 1 + 3 (PR-10 protein+ non-pecific lipid transfer protein type 1, strawberry), Gly m 4 (soy, PR-10 protein), Jug r pollen (walnut pollen), Mal d 1 (apple, PR-10 protein), Table S1.

In Table 3, we search the unique combinations (the level of specific IgE in Classes 3 or 4), such as to cover the largest possible set of patients. It is calculated according to TURF (total unduplicated reach and frequency). We found 75 allergen reagents (molecular components) with the level of specific IgE in Classes 3 and 4, with this positivity in 6 and more patients. We also show the number of positive results of specific IgE to 75 selected molecular components and allergen extracts in Classes 0, 1, 2 (Table 3).

In Table 4, we show 81 allergen reagents (allergenic extracts and molecular components) with these unique combinations (the level of specific IgE in Classes 3 or 4 calculated according to TURF) with positivity at least in 5 patients. We performed the detailed statistical analysis to determine the relationship between the positive results of specific IgE (in Classes 3 and 4) of these allergen extract and molecular components to the occurrence of bronchial asthma, allergic rhinitis, the severity of AD, the onset of AD, family history and duration of eczematic lesions. The levels of significance (*p*-value) are calculated in the chi-square test (at an expected minimum frequency of at least 5) or in Fisher’s exact test.

We show a significant relation to examined parameters *(p-*value < 0.05).

**Severity of AD**

Blo t 5—Storage mites, Blomia tropicalis
Aca s—Storage mites, Acarus siro
Gly d 2, Lep d 2—Storage mites, NPC2 family
Can f 1, Can f 2, Can f 4 and Can f 6—Lipocalins
Fel d 1, Ory c 3—Uteroglobin
Mala s 11—Mn superoxide dismutase
Asp f 6, Bla g 9, Der p 20, Pen m 2—Arginine kinase
Fag s 1, Mal d 1, Cor a 1.0401, Cor a 1.0103—PR-10 proteins
Cyn d 1, Lol p 1, Phl p 1—Beta-expansins
Phl p 2—Expansin
Der p 21, Der p 23—Peritrophin-like domain.
Secc pollen—Cultivated rye
Cra c 6—Troponin C (North Sea shrimp)
Hom g—Lobster
Ara h 6-2S Albumin, Peanut, Ara h 1-Cupin (Vicillin-type, 7S globulin), Peanut

**Bronchial asthma**

Alt a 6—Enolase, Alternaria alternata
Lep d 2, Der p 2, Der f 2—NPC2 family
Der p 1—Cystein protease
Der p 21, Der p 23—Peritrophin-like protein domain
Der p 5, Der p 7—European house dust mite
Loc m—Migratory locust
Phl p 1—Beta expansin
Phl p 2—Expansin
Secc pollen—Cultivated rye
Tyr p—Storage mite

**Allergic rhinitis**

Api g 1, Bet v 1, Cor a 1.0103, rCor a 1.0401, Dau c 1, Fag s 1—PR-10 proteins
Cyn d 1—Beta expansin
Can f—1 Lipocalin
Fel d 1—Uteroglobin
Der f 1—Cystein proteasa
Phl p 5, Phl p 6—Grass group 5, 6 Timothy.

**Onset of AD**

Can f 1, Can f 2, Can f 4, Cav p 1, Fel d 4, Fel d 7—Lipocalins
Phl p 1—Beta expansin
Phl p 2—Expansin
Fel d 1—Uteroglobin
Der p 21, Der p 5—House dust mites and
Asp f 6—Mn superoxide dismutase, Aspergillus fumigatus

**Family history**

Der f 2, Der p 2—NPC2 family, house dust mite
Fel d 1—Uteroglobin
Fel d 4, Mus m 1—Lipocalins
Mala s 5—Malassezia sympodialis
Rat—extract specific IgE of Rat

**Persistent eczematic lesions**

Aca s, Blo t 5, Tyr p—Storage mites
Pen m 2, Der p 20, Bla g 9—Arginine kinase
Asp f 6—Mn superoxide dismutase, Aspergillus fumigatus
Der f 2, Der p 2—NPC2 family, house dust mite
Gly d 2 and Lep d 2—NPC2 family, storage mite
Der p 5, Der p 7, Der p21—Molecular components from house dust mites
Der p 23—Peritrophin-like domain
Can f Fel d 1-like—Uteroglobin
Cor a 1.0103, Fag s1—PR-10 proteins
Phl p 1—Beta expansin
Phl p 2—Expansin

In Table 5, we show the detailed comparison of sensitization to allergen reagents according to the level of specific IgE (Classes 0, 1, 2, 3, 4) in patients suffering from mild, moderate, and severe form (comparison of column proportions, two-sides test). We show only the significant relation (*p*-value < 0.05). According to these results, in patients suffering from a mild form, we observe in general low sensitization to examined allergen reagents.

### 2.1. Comparison of Mild and Severe Form of AD

The negative level of specific IgE (Class 0) was observed with the significantly higher occurrence in patients suffering from mild form in comparison to patients suffering from severe form to these molecular components:

Acas—storage mite, Api g 1, Mal d 1—PR-10 protein, Fra a 1 + 3—PR-10 protein+ non-specific lipid transfer protein type 1, strawberry, Bla g 9—German cockroach, Can f 1, Equ c 1, Fel d 4—lipocalins, Lep d 2—NPC2 family, Tyr p—storage mite, Cyn d 1—Beta expansin, Pen m 2—arginine kinase

The level of specific IgE in Class 4 (very high level of sIgE) was observed with the significantly higher occurrence in patients suffering from a severe form in comparison to patients suffering from a mild form to these molecular components:

Bet v 1—PR-10 protein, Bla g 9—German cockroach, Can f 6—lipocalin, Cla h 8—manitol dehydrogenase, Cladosporium herbarum, Der p 20—arginine kinase, Lol p 1, Phl p 1—Beta expansin, Phl p 5—grass group 5

### 2.2. Comparison of Mild and Moderate Forms of AD

The negative level of specific IgE (Class 0) was observed with a significantly higher occurrence in patients suffering from the mild form in comparison to patients suffering from moderate form to molecular component: Can f 1—lipocalin.

The level of specific IgE in Class 2 (moderate level of sIgE) was observed with a significantly higher occurrence in patients suffering from a moderate form in comparison to patients suffering from a mild form to these molecular components: Bet v 1, Fag s 1—PR-10 protein.

### 2.3. Comparison of Moderate and Severe Forms of AD

The negative level of specific IgE (Class 0) was observed with the significantly higher occurrence in patients suffering from a moderate form in comparison to patients suffering from severe form to these allergen extracts and molecular components:

Acam—acacia, Acas—storage mite, All c—onion, Amar—redroot pigweed, Api g 1—PR-10 protein

Ara h 1—cupin (vicillin-type, 7S globulin), Ara h 2—conglutin (2S albumin), Ara h 3—cupin (legumin-type, 11S globulin, glycinin), Asp f 1—mitogilin family, Asp f 6—Mn superoxidase dismutase, Bet v 2—profilin,

Bla g 4—German cockroach, Bla g 9—arginine kinase, German cockroach, Blo t 21—Blomia tropicalis,

Blo t 5—tropomyosin, Blomia tropicalis, Cap a—chili, bell pepper, Cla h—Cladosporium herbarum, Car p—papaya, Cla h 8—short-chain dehydrogenase, Cra c 6—troponin C, North Sea shrimp, Cuc m 2—muskmelon, profilin, Cup a 1—cypress, Pectate lyase, Der p 20—arginine kinase, Dols pp, Equ c 1—lipocalin, Equ c 3—serum albumin, Fel d 4—lipocalin, Frae—ash, Frae 1—ash, Ole e 1 family, Gad m—cod, Gal d 1—ovomucoid, Gal d 2—ovoalbumin, Gal d 3—ovotransferin, Gal d 5—serum albumin Gal d white—egg white, Gal d yolk—egg yolk, Hel a—sunflower, Hev b 8—profilin, latex, Hor v—barley, Chea—quinoa, quinoa,

Jug r pollen—English walnut, Lup a—white lupine, Mala s 11—manganese superoxide dismutase, Mala s 6 cyclophilin, Man i—mango, Mer a 1—profilin, Mus a—banana, Ory c 2—lipophilin, Pen m 2—arginine kinase, Per a—American cockroach, Per a 7—tropomyosin, Pers a—avocado, Phl p 12—prophylin, Pho d 2—profilin, date palm, Rudspp—mussel, Sacc—sacharomyces cerevisiae, Sal k 1—pectin methylesterase, Sco s—Atlantic makrel, parvalbumin, Secc flour, Ses i—sesame, 2S albumin, Sol t—potatoes, Tri fo—seeds, Tyr p—storage mite, Urt d, Ves v, (see Table 5—moderate form of AD marked “C“).

The level of specific IgE in Class 3 and 4 (high and very high level of sIgE) was observed with the significantly higher occurrence in patients suffering from a severe form in comparison to patients suffering from moderate form to these molecular components:

Amba—Ragweed, Ara h 1—Cupin (Vicillin-type), 7S globulin, Alt a 6—Enolase, Alternaria alternata, Asp f 6—Mn superoxide dismutase, Aspergillus fumigatus, Bla g 9—German cockroach, Can f 2—lipocalins, Can f 6—lipocalins, Cyn d 1—Beta expansin, Der p 20—arginine kinase, Der p 21, Der p 23—peritrophin-like domain, Fel d 4 — lipocalin, Gal d 2—ovoalbumin, Gly d 2—NPC2 family, storage mite, Gly m 4—PR—10 protein, soy, Hev b 8—profilin, latex, Lol p 1—Beta expansin, Mala s 11—manganese superoxide dismutase, Phl p 2—expansin

According to statistical methods TURF and best reach and frequency by group size, we selected other slightly smaller sets of allergen extracts and molecular components that cover 75% of patients and 52% of patients, (Table 6).

**Figure 1:**
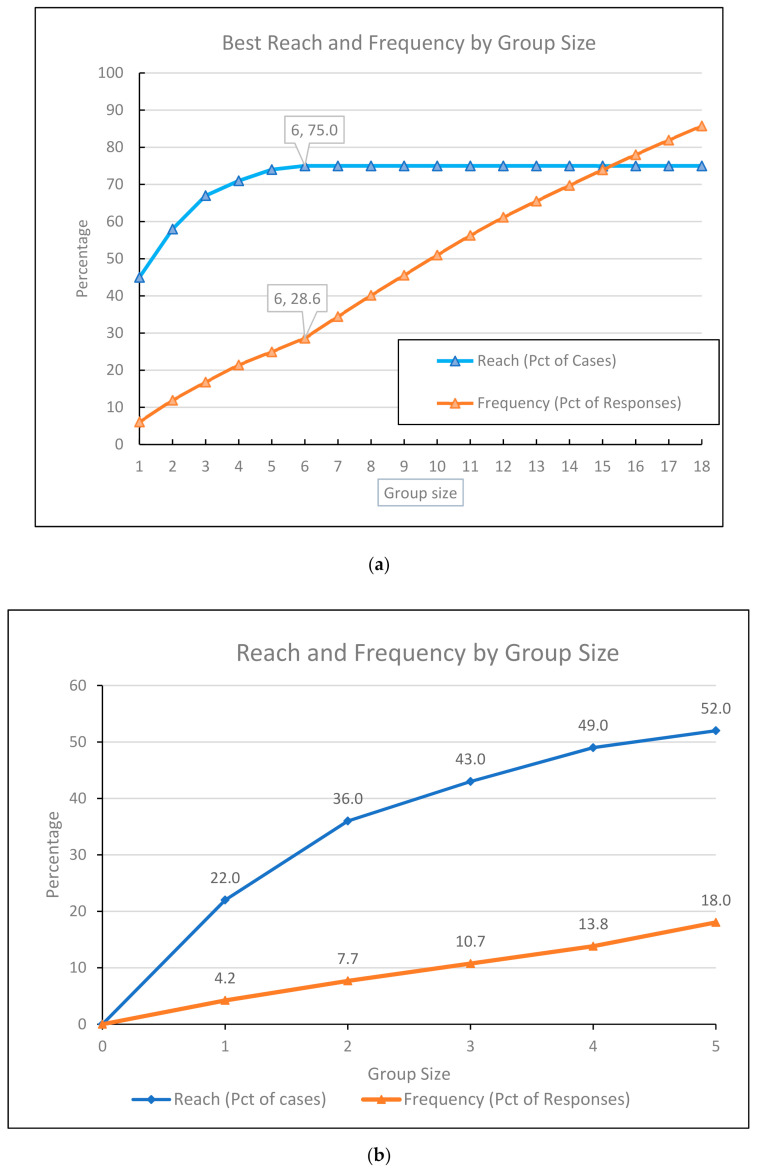
(**a**) Best reach and frequency by group size-18 molecular components. (**b**) Best reach and frequency by group size–5 molecular components).

We found leading 18 molecular components with a high level of specific IgE (Class 3) and very high level of specific IgE (Class 4). The level of specific IgE in Classes 3 and 4 to these molecular components reached 75 patients (75.0%), the frequency of positivity was at a rate of 632, Table 6, the Figure 1a. The detailed analysis of this statistic method we show in Table S2. We found these 18 molecular components and allergens extracts as the leading allergens: Aln g 1 (alder, PR-10 protein), Alt a 1 (Alternaria alternata), Bet v1 (birch, PR-10 protein), Can f 1 (lipocalin, dog), Cor a1.0103 (hazel pollen, PR-10 protein), Cor a1.0401 (hazelnut, PR-10 protein), Cora_pollen, Der f 2 (house dust mite, NPC2 family), Der p 2 (house dust mite, NPC2 family), Fag s 1 (European beech, PR-10 protein), Fel d1 (cat, uteroglobin), Lol p 1 (rye grass, Beta expansin), Phl p 1 (Timothy, Beta expansin), Phl p 2 (Timothy, expansin), Phl p 5_0101, Phl p 6, (Timothy, grass group 5/6), Secc_pollen (cultivated rye), Der p 23 (peritrophin-like protein domain, house dust mite).

We found smaller sets of leading five molecular components with a high level of specific IgE (Class 3) and a very high level of specific IgE (Class 4). The level of specific IgE in Classes 3 and 4 to these molecular components reached 52 patients (52.0%), the frequency of positivity was at a rate of 94, Table 6, the Figure 1b. The detailed analysis of this statistic method we show in Supplementary Table S2 We can observe that 52% of AD patients reached a positive level of specific IgE to these molecular components: Fel d 7 (lipocalin, cat) Art v (mugwort), Bla g 9 (arginine kinase, German cockroach), Cyn d (Bermuda grass) and Can f 4 (lipocalin, dog).

## 3. Discussion

The purpose of our study was to evaluate the results of specific IgE to allergen reagents (117 allergenic extracts and 178 molecular components) in patients suffering from atopic dermatitis with the new multiplex assay (ImmunoCAP ALEX2 examination). There are several commercially available immuno-solid-phase multiplex allergen arrays: the Thermo Fisher ImmunoCAP ISAC (Immuno-solid-phase Allergen Chip), which contains 112 allergens from 51 allergen sources; the new ImmunoCAP ISAC 112i, with 112 components from 48 allergen sources, the MADx Allergen Explorer (ALEX; containing 282 allergens: 156 extracts and 126 components) and the Euroline microstrips [19,23,24].

According to our knowledge, there are no studies dealing with this examination ALEX2 in patients suffering from atopic dermatitis. Regardless of the levels of positive specific IgE, the highest sensitization rate to grass-species specific component Phl p 1 (Timothy, Beta expansin) reached 57% of patients. The second most frequent sensitization rate to components of birch Bet v 1 (PR-10 protein), Lol p 1 (rye grass, Beta expansin), and Secc pollen were observed in 53% of patients. A high sensitization rate is observed to cross-reactive PR-10 proteins, to Beta expansin from Bermuda grass, to molecular components of Timothy, to molecular components of NPC2 family, and uteroglobin. We performed our assessment also with regard to the levels of specific IgE and we found 81 allergen reagents (molecular components and allergens extracts) for the detailed analysis. According to this analysis, we confirmed that the severity of AD is in significant relation to the sensitization to molecular components of storage mites, lipocalins, arginine kinase, uteroglobin, Mn superoxide dismutase (Mala s 11), PR-10 proteins, Der p 21, Der p 23—peritrophin-like domain and to Secc pollen. From food allergen, we confirmed only the role of Cra c 6 (Troponin C (North Sea shrimp), Pen m 2 (Arginin kinase, shrimp) and Hom h (Lobster) in the severity of AD. In subgroup of patients suffering from bronchial asthma, the significant role plays molecular components from European house dust mites and storage mites, Alt a 6 and Phl p 1. In patients suffering from rhinitis, the important role plays PR-10 proteins, lipocalin, and uteroglobin and grass group 5, from house dust mites, the role plays only Der f 1—cysteine protease. In patients with the onset of AD under 5 years of age, there is a significantly higher occurrence of sensitization, mainly to lipocalins and uteroglobins, and to Asp f 6. There is a significant relationship between the positive family history about atopy and sensitization to molecular components from house dust mites, uteroglobin, lipocalin, and Mala s 5. A significant relation was confirmed between the persistent eczematic lesions and sensitization to molecular components of storage mites, house dust mite (NPC2 family), Der p 23, Asp f 6, and Phl p 1.

We also determined the leading 18 molecular components with high (Class 3) and very high level of specific IgE (Class 4) with positive results in 75.0% of patients. The most important role play molecular components from PR-10 proteins (Aln g 1, Bet v 1, Cor a1.0103, Cor a1.0401, Fag s 1), lipocalin (Can f 1), NPC2 family (Der f 2, Der p 2), uteroglobin (Fel d 1), Beta expansin (Lol p 1, Phl p 1), molecular components from Timothy, cultivated rye (Secc pollen), Peritrophin-like protein domain Der p 23 and Alt a 1 from Alternaria alternata. Although the sensitization rate in the whole group of patients to Alt a 1 is 26%, the high and very high level of specific IgE to Alt a 1 reached a significant number of patients although without significant relation to bronchial asthma, allergic rhinitis, or severity of AD.

Our study shows that 52% of AD patients reached a high and very high level of specific IgE to other lipocalins such as Fel d 7 and Can f 4, to Art v (mugwort), Bla g 9 (Arginine kinase, German cockroach), and to Cyn d (Bermuda grass). As Bermuda grass pollen, as well as Bahia grass, are not present in our region, possible cross-reactivity with β-expansins from other grasses could be the explanation for the results with high sensitization to Cyn d 1 and Pasn in our study.

In our previous study with ISAC, we also found these leading molecular components: Der f 1, Der p 1 (cysteine protease), Mal d 1 (PR-10 protein), Mus m 1 (Lipocalin) Phl p 4 (berberine—bridge enzyme), (21, 22). On the other hand, Der p 23 is not included in ISAC, but Der p 23 is one of the most important molecular components in ALEX2. The level of specific IgE to Der p 23 in Class 4 was recorded with the significantly higher occurrence in patients suffering from a severe form of AD and as well in patients suffering from bronchial asthma. The close association of Der p 23 with fecal pellets may be one reason why it represents a major allergen and exhibits high allergenic activity. According to Weghofer, the Der p 23—specific IgE levels of the tested patients were comparable to the IgE levels to Der p 1 and Der p 2. Because of its high frequency of IgE recognition and allergenic activity, Der p 23 must be considered as an essential component for the diagnosis and specific immunotherapy of house dust mite allergy, as was confirmed in our study [25]. Other molecular components of house dust mites and storage mites play an important role in atopic dermatitis patients, according to our results in ALEX2 Allergy Explorer. The level of specific IgE to Der f 2, Der p 2 (house dust mites, NPC2 family) are significantly higher in the subgroup of patients suffering from bronchial asthma, to Gly d 2 and Lep d 2 (storage mites, NPC2 family) in patients with persistent eczematic lesions. The main species from house dust mites are D. pteronyssinus, D. farinae, Euroglyphus maynei. Storage mites (include Lepidoglyphus destructor, Blomia tropicalis, Glycyphagus domesticus, Tyrophagus putrescentiae, Acarus siro, Aleuroglyphus ovatus, Suidasia medanensis, and Thyreophagus entomophagus) are global pests of stored food products of increasing medical and economical impact. In agricultural environments, they cause occupational allergies in farmers and grain handlers. Storage mites are also found in house dust from rural and urban dwellings and are important contributors to the allergen content, which expands their clinical significance. The storage mites belong to the Acaridae and Glycyphagidae families. Their prevalence varies depending on the geographical location. D. pteronyssinus and D. farinae have been shown as the common allergens worldwide, whereas, Blomia tropicalis is the predominant mite species in tropical and subtropical regions, and in some areas, it coexists with D. pteronyssinus [26,27]. Our results confirmed the significantly higher sensitization to Blo t 5 (Blomia tropicalis, mites group 5) and to Aca s (Acarus siro) in patients suffering from severe and persistent forms of AD. Carvalho et al.l. demonstrated that rBlo t 5 and rBlo t 21 were antigenic for the Blomia tropicalis-sensitized population, and they showed that the IgE reactive to these allergens had less cross-reactivity with Ascaris lumbricoides extract than anti-Blomia tropicalis extract IgE, as assessed by an IgE-binding inhibition assay. Thus, rBlo t 5 and rBlo t 21 expressed in E. coli may be potential candidates to be used in a pool of different recombinant allergens for improving serodiagnosis assays of allergy to Blomia tropicalis [28]. Acarus siro produced a high level of alpha-amylase activity attributed to Aca s 4. Homology modeling of Aca s 4 revealed a structural change in the chloride-binding site that may account for this activation pattern. Aca s 4 was recognized by IgE from house dust mite-sensitive patients, and potential epitopes for cross-reactivity with house dust mite group 4 allergens were found. [29].

In 2014, it was reported that IgE antibodies to Der p 11 are more common in sera from patients with atopic dermatitis [30]. Thus, sensitization to this allergen may reflect the fact that the eczematous skin allows easy penetration of allergens even with molecular weight as high as 100,000. Der p 11 is a protein of 874 amino acids with a deduced molecular mass of ~103 kDa, and it shows high sequence identities (>85% sequence identity) with paramyosins from house dust mites, itchy mites, and tropical mites [30]. According to our results, the sensitization to molecular component Der p 11 was confirmed only in 3 patients from the whole study (3%). Two of these patients suffer from a severe form of AD and one patient from a moderate form of AD.

Molecular component Mal d 1 (PR-10 protein, apple) was confirmed as the leading molecular component in our previous study in ISAC, but not in ALEX2 Allergy Explorer. In ALEX2, we found a moderate level of specific IgE in 17% of patients, a high level in 10% of patients, and a very high level in 13% of patients; the significant relation between sensitization to Mal d 1 and severity of AD was confirmed. In ISAC we confirmed high sensitization to Phl p 4 (in 52% of patients). In ALEX2 there is no included examination of specific IgE to Phl p 4. Natural Phl p 4 contains cross-reactive carbohydrate determinants (CCD), which may lead to IgE cross-reactivity with a wide range of plants and plant products. This explains why in several epidemiological studies IgE positivity to Phl p 4 scores over 90% of the grass pollen-allergic patients. However, when the recombinant version of the molecules is used for assays, over 50% of the positivity is not confirmed anymore [9]. Although recombinant allergens expressed in *E. coli* lack glycosylation, the natural purified allergens have the same sugars as their natural counterparts [30]. Highly glycosylated allergens induce the production of sIgE against the sugar moiety (CCD), which can be responsible for cross-reactivity. Six highly glycosylated allergens are in their natural purified form in the ImmunoCAP ISAC: walnut nJug r 2, Bermuda grass nCyn d 1, Timothy grass nPhl p 4, Japanese cedar nCry j 1, Arizona cypress nCup a 1, and plane nPla l 2. [31]. It is not possible to determine whether IgE to these 6 allergen components is directed to the protein or the carbohydrate side chain, so ruling out the presence of sIgE against CCD is important, especially when other markers of genuine sensitization to the same source are lacking.The greatest value of ALEX is the provision of a CCD inhibitor, which reduces clinically irrelevant cross-sensitization and has a direct impact on primary sensitization information [19]. In ALEX2, from 81 allergen reagents recorded with high and very high level of specific IgE, majority of molecular components are recombinant; only Ara h 1, Ara h 6, Lol p 1, and Ole e 1 are in their natural purified form.

Patterns of sensitization vary depending on the geographical area. The knowledge of local molecular epidemiology is essential for guiding allergists in choosing the components to test in their population and interpret the results properly [32]. The leading molecular components from inhalant allergens are in our study PR—10 Proteins: Aln g 1 (alder), Bet v 1 (birch), and Cor a1.0103 (Corylus avelana) and Cor a pollen. We compared the results of our study with the botanical characteristic of our region. Based on data from the nearest phenological station Běleč nad Orlicí (241 m above sea level), 50°12′ N, 15°56′ E), which is located in the immediate vicinity of Hradec Králové (East Bohemia region in Czech Republic), the following species are present from important pollen allergens: owing to the sandy substrate, white birch (Betula pendula) and wood pine (Pinus sylvestris) predominate, as well as common hazel (Corylus avellana). There is also abundant sticky alder (Alnus glutinosa), willow (Salix caprea), hornbeam (Carpinus betulus), summer oak (Quercus robur), spruce (Picea excelssa), deciduous larch (Larix decidua), Heartworm (Tilia cordata). For trees flowering in the phenological pre-spring (hazel and alder), the shift is 11 to 29 days. The most important pollen allergen, white birch blooms on average more than 10 days earlier. Birch and other related trees of the families Betulaceae and Fagaceae (alder, hazel, oak, hornbeam, chestnut, and beech) constitute the birch homologous group [32]. This grouping is primarily based on the extensive IgE cross-reactivity of allergen homologs to the major birch allergen Bet v 1. Birch pollen is the most dominant tree pollen in Northern and Central Europe and is a major cause of allergic rhinitis and, possibly, asthma symptoms. Over the last few decades, levels of birch pollen have risen, and the period of exposure has increased due to climate changes. Subsequently, the prevalence of birch pollen sensitization has also increased. The cross-reactivity and sequential pollen seasons within the birch homologous group create a prolonged symptomatic allergy period beyond birch pollen alone [32]. Furthermore, many plant food allergens contain homologs to Bet v 1, meaning that the majority of patients with birch pollen allergy suffer from secondary pollen food syndrome (PFS). As a result, the negative impact on health-related quality of life (HRQoL) in patients allergic to birch pollen is significant. According to our results, in the subgroup of patients suffering from allergic rhinitis, the important role plays mainly PR-10 proteins.

We compared our results with other studies from the Middle-European region in the point of view of the outcomes describing the sensitization patterns to molecular components [33,34,35]. Panzner et al. investigated 1255 sensitized patients, with a mean age of 29 years, and with the following diagnoses: chronic rhinitis (73%), bronchial asthma (41%), atopic dermatitis (34%), urticaria or edema (19%), and/or anaphylaxis (11%), [33,34,35]. Our results are in agreement with Panzner’s hypothesis [33,34,35] that grasses (Phl p 1) and *Betulaceae* (Bet v 1) components comprised the vast majority of pollen sensitizations in the condition of the Middle-European region. On the other hand, in our study, the sensitization to animal allergen molecules and to mite molecular allergens was significantly higher. The explanation can be in the fact that we included patients suffering from atopic dermatitis; in Panzner’s study, atopic dermatitis patients represent only 34% of patients. Our results may demonstrate the significance of disturbed epidermal barrier, resulting in increased transepidermal water loss and penetration of allergens, irritants, and microbes.

We confirmed the relation between the severity of AD and the sensitization to lipocalins and uteroglobins; the association was also confirmed with the onset of AD as well with the allergic rhinitis, but not with bronchial asthma. The homology between different dog and cat allergens such as albumins and lipocalins explains the cross-sensitization between them and allergens from other mammals and the presence of simultaneous sensitization to dogs, cats, and other mammals regardless of whether there is or is not direct exposure to all of them [36,37,38]. Some of these antigens cause clinically relevant cross-reactivity between different animals. The most significant cross-binding patterns between allergens of cats, dogs, and other mammals are with lipocalins and Can f 5. Lipocalins have amino acid sequences with up to 60% identity with Can f 6 (dog), Equ c 1 (horse), Fel d 4 (cat), Ory c 4 (rabbit), and Mus m 1 (mouse). Can f 5 shows a certain homology with prostate-specific antigen, which is also in the kallikrein family. It has been speculated that prior sensitization to Can f 5 from dogs could be associated with a greater propensity for developing allergic reactions to human seminal fluid [36,37,38]. Finally, Can f 7, an NPC2-like protein that is homologous with the NPC2 components of house dust mites, has been identified in dogs [36,37,38]. Sensitization to certain allergens seems to be associated with the severity and persistence of clinical symptoms, and sensitization to more than 1 allergen or sensitization to albumins seems to be associated with more severe respiratory symptoms [39]. Exposure before and around birth to dog dander or to dust from cow barns is regarded as protective against allergies and asthma. However, if sensitization does occur, multiplexing could be helpful to identify the extent of risk for the individual patient to define avoidance or AIT strategies [40] and to identify other animals that may be a source of clinically relevant cross-reacting allergens despite no prior exposure. Sensitization to Can f 1 and Fel d 1 and polysensitization to cat and dog allergens during childhood have been associated with the development of subsequent allergy to cats and dogs [40,41]. In 259 children sensitized to cats, co-sensitization to Fel d 1 and Fel d 4 was associated with a risk for asthma; for dog sensitization, IgE to Can f 5, 1, and 2 were the most significant risk factors [40,41]. According to our results, the sensitization to lipocalins and uteroglobins is significantly higher in patients with the onset of AD under 5 years of age.

Mitterman et al. [42] characterized in their study the specificities of IgE reactivity in patients with AD to a broad panel of exogenous allergens, including microbial and human antigens. In their study, adult patients with AD were grouped according to the SCORAD index, into severe (*n* = 53) and moderate AD (*n* = 126). Sensitization to cat allergens occurred most frequently, followed by sensitization to birch pollen, grass pollen, and to the skin commensal yeast *M*. *sympodialis*. Patients with severe AD showed a significantly higher frequency of IgE reactivity to allergens such as cat (Fel d 1) and house dust mite (Der p 4 and 10), there were no significant differences in the frequencies of IgE reactivity to the grass pollen allergens Phl p 1, 2, 5 and 6 between the two AD groups, and the IgE reactivity profile of patients with severe AD was more spread toward several different allergen molecules as compared to patients with moderate AD (37). According to Mitterman’s study, it would suggest that grass pollen allergens are less important as trigger factors for AD compared to birch pollen and indoor allergens in their studied population [42].

Röckman [43] et al. investigated the pattern of food sensitization in 211 adults with AD in relation to AD severity using multiplexed allergen microarray. Specific IgE levels were routinely measured using a microarray immunoassay (Immuno Solid-phase allergen Chip (ImmunoCAP ISAC^®^, VBC Genomics, and Phadia)) with 103 allergens. Sensitization to PR-10 related food allergens occurred most frequently (63.5%) and was independent of AD severity. Of all plant food allergens, only sensitization to *nAra h 1* was significantly more frequent in patients with severe AD. In the total group, 75 (35.5%) patients with AD showed sensitization to any animal food allergen. The percentage was significantly higher in patients with severe AD (51.4%) compared to patients with mild/moderate AD (27.7%) [33].

According to our results in evaluating food allergens, we confirmed the important role of molecular components from egg, soy, cod, shrimp, peanuts, and cross-reactive components from PR-10 proteins.

## 4. Materials and Methods

Aim of this study is to analyze the sensitization profile to 295 allergen reagents (117 allergenic extracts and 178 molecular components) in patients suffering from atopic dermatitis with the use of ALEX2 Allergy Explorer.

(a) To determine the sensitization to these allergenic extracts and molecular components in the whole group of AD patients and to show the level of specific IgE in the whole group of AD patients;

(b) To find allergenic extracts and molecular components with a high and very high level of specific IgE to cover the largest possible set of patients;

(c) To evaluate the relation between the examined parameters (bronchial asthma, allergic rhinitis, severity of AD, onset of AD, family history, duration of eczematic lesions) and the results of specific IgE in ALEX2 Allergy Explorer.

### 4.1. Statistical Analysis

We performed the deep statistical analysis with the use of several methods of statistics.

With the method of total unduplicated reach and frequency (TURF, IBM SPSS Statistics, version 27)**,** we search the unique combinations (here attributes with the desired property—the level of specific IgE in Classes 3 or 4), such as to cover the largest possible set of given elements (here patients = reach) for which some occurrences have been detected and at the same time the largest number of occurrences of the searched property on given attributes (frequency).

### 4.2. Commentary on Statistical Evaluation

Two opposing requirements come along with the availability of the large set of attributes describing the state of interest. They follow from the necessity to keep the balance between predictive precision (the accuracy) and simplification of decision rules, based on the selection of an appropriate set of attributes supporting the decision. The goal of the total unduplicated reach and frequency analysis is to detect the set (combination) of attributes from the 295 available outcomes on the given group of 100 persons suffering from a specific disease. The selected sub-set of attributes has to cover most of the positive outcomes (3 + 4) observed on most objects.

The computation complexity of this optimization problem increases with the size of the set of candidate attributes that can potentially detect the majority of positive outcomes on most persons.

The setting of the threshold for minimum expected responses in the analysis limits the dimension of the final solution (attributes with occurrence below the threshold are not evaluated). The process starts with the small set (usually one attribute with the highest occurrence) and in the next step takes into consideration only attributes (from candidate set) not covered yet (unduplicated search). As a result, the sets of sub-optimal solutions are presented along with their frequency and reach. Frequency is the ratio of positive outcomes on the set of selected attributes to the total of positive outcomes in the candidate set. The reach is the proportion of individuals with an outcome (3 + 4) on the given set.

There can be no unique optimal solution. Possible sub-optimal solutions usually have similar properties (measured by reach and frequency).

In medical research, the method can help to detect the combination (the set) of the most frequent attributes presenting the positive outcomes when tens of attributes have to be evaluated simultaneously. As a complementary step, the following statistical analysis has to be performed on the sets presenting similar reach (ratio of objects covered by the set) and frequency (ratio detected values of interest on the set) and have to combine results given on different sets of candidate attributes.

We evaluate the relation between the sensitization to allergen reagents (found in TURF) and the examined parameters, such as the occurrence of asthma bronchiale, allergic rhinitis, severity of AD, onset of AD, family history, and duration of eczematic lesions. The levels of significance (*p*-value) are calculated in chi-square test (at an expected minimum frequency of at least 5) or in Fisher’s exact test.

We evaluate the relation between the sensitization to allergen reagents (according to the level of specific IgE in Classes 0, 1, 2, 3, 4) and the severity of AD (mild, moderate, and severe form of AD) with two-sided tests. For each significant pair, the key of the category with the smaller column proportion appears in the category with the larger column proportion. The significance level for upper case letters (A, B, C):0.05. Explanation: „a“—this category is not used in comparisons because its column proportion is equal to zero or one.

According to TURF, we select other slightly smaller sets that cover 75% and 52% of patients (best reach and frequency by group size). We show the detailed tables together with graphs.

When introducing the test into the clinical examination, we also examined 15 healthy volunteers—blood donors (equivalent to age, male and female representation). All of these blood donors had in the multiplex examination (ALEX2 Allergy Explorer) the specific IgE negative, expressed as Class 0 (<0.3 kUA/L)

### 4.3. Patients and Methods

In the period 2018–2020, 100 patients suffering from atopic dermatitis at the age of 14 years and older were examined. All these patients live in the East Bohemia region in the Czech Republic in middle Europe. There are 551,000 inhabitants, and the area of this region is 4759 square km. All these patients were examined in the Department of Dermatology, Faculty Hospital Hradec Králové, Charles University, Czech Republic. The diagnosis of atopic dermatitis was made with the Hanifin-Rajka criteria [44]. Exclusion criteria were systemic therapy (cyclosporin, systemic corticoids, biological therapy), pregnancy, breastfeeding. Patients with atopic dermatitis having other systemic diseases were excluded from the study as well. The complete dermatological and allergological examination was performed in patients included in the study. This study was approved by the ethics committee of the Faculty Hospital Hradec Králové, Charles University of Prague, Czech Republic.

### 4.4. Dermatological Examination

The complete dermatological examination was performed in patients included in the study. The severity of atopic dermatitis was scored in agreement with SCORAD, with the assessment of topography items (affected skin area), intensity criteria, and subjective parameters. To measure the extent of atopic dermatitis, the rule of nines was applied on a front/back drawing of the patient’s inflammatory lesions. The extent was graded 0–100 points. The intensity part of the SCORAD index consists of six items: erythema, edema/papules, excoriations, lichenification, crusts, and dryness. Each item was graded on a scale of 0–3. The subjective items included daily pruritus and sleeplessness. Both subjective items were graded on a 10-cm visual analog scale, and the maximum subjective score was 20 points. All items were filled out in the SCORAD evaluation form. The SCORAD index formula was: A/5 + 7B/2 + C. In this formula, A is defined as the extent (0–100 points), B is defined as the intensity (0–18 points), and C is defined as the subjective symptoms (0–20 points). The severity of atopic dermatitis is evaluated with SCORAD as a mild form to 20 points, as moderate over 20 to 50 points, as a severe form over 50 points [45]. The evaluation of the SCORAD score was performed every two months during the study [45].

### 4.5. Examination of SpecificIgE to Molecular Components

The serum level of the sIgE was measured by the components resolved diagnostic microarray-based sIgE detection assay ImmunoCAP ALEX—ALEX2 Allergy Explorer [20,46,47]. It is based on state-of-the-art proprietary nano-bead technology. This new array contains 295 allergen reagents (117 allergenic extracts and 178 molecular components), with a large majority of aeroallergen families and cross-reactive food allergens being represented. The ALEX2 Allergy Explorer measuring range for specific IgE is 0.3–50 kU_A_/L (quantitative) and for total IgE is 1–2500 kU/L (semiquantitative). The sampling requirement is 100 µL serum or plasma. The results are expressed as Class 0 (<0.3 kU_A_/L), Class 1 (0.3–1 kU_A_/L), Class 2 (1–5 kU_A_/L), Class 3 (5–15 kUA/L), and Class 4 (>15 kU_A_/L). ALEX is commercially available, having attained CE certification, which assures that the quality of the assay, regarding LoD, precision, and repeatability, as well as specificity and linearity, is in line with in vitro diagnostic features. There is no significant interference from high total IgE, hemoglobin, bilirubin, or triglycerides. A flexible Raptor analysis software (specifically designed for ALEX^®^) allows analyzing tailormade allergen panels, as considered fit for clinical needs (multiplex on-demand), [20].

Initially, allergens are coupled to activated nanoparticles for coupling individual and combinatorial optimization. Each allergen is attached, reflecting its biochemical properties and specific requirements for stability, thereby preserving the full epitope complexity. The nanoparticles multiply the surface of the solid phase presenting the allergen during the immunoassay, enabling highly sensitive detection. In the next step, the allergen-bearing nanoparticles are deposited onto a solid-phase matrix, forming a macroscopic array of individual assay parameters. The different allergens and components, spotted onto a nitrocellulose membrane as immunosorbent in a cartridge chip, are incubated with 0.5 mL of a 1:5 dilution of serum under agitation, the serum diluent containing a cross-reactive carbohydrate determinants (CCDs) inhibitor. After incubation for 2 h, the chips are extensively washed. A pretitered dilution of anti-human IgE labeled with alkaline phosphatase is added and incubated for 30 min. Following another washing cycle, the enzyme substrate is added, and after a few minutes, the reaction is complete. After the membranes are dried, the quantification of this colorimetric enzyme assay is performed with an easy-to-use and affordable image explorer. The image acquisition and analysis of a single test took only a few seconds. The assay time is 3.5 h, and tests per run are up to 50 per operator, with manual processing [18,19].

When introducing the test into the clinical examination, we also examined 15 healthy volunteers—blood donors (equivalent to age, male and female representation). All of these blood donors had in the multiplex examination (ALEX2–Allergy Explorer) the specific IgE negative, expressed as Class 0 (<0.3 kUA/L). This study was approved by the ethics committee of the Faculty Hospital Hradec Králové, Charles University of Prague, Czech Republic.

### 4.6. Bronchial Asthma

The diagnosis of bronchial asthma (AB) was determined according to the guidelines of the Global Initiative for Asthma (GINA) at the allergy outpatients clinic of the Institute of Clinical Immunology and Allergology, Faculty Hospital Hradec Kralove, Czech Republic (Global Initiative for Asthma. Global Strategy for asthma management and prevention—Update 2015. www.ginasthma.com (accessed on 25 January 2021)).

### 4.7. Allergic Rhinitis

The evaluation of allergic rhinitis (AR) was made according to the allergy testing and personal history of the Institute of Clinical Immunology and Allergology, Faculty Hospital Hradec Kralove, Czech Republic [48]. AR was defined as a process that included 3 cardinal symptoms: sneezing, nasal obstruction, and mucus discharge. Symptoms occur with allergen exposure in the allergic patient [48].

### 4.8. The Evaluation of Duration of Atopic Dermatitis

The atopic dermatitis lesions were evaluated as persistent or occasionally, according to the dermatologist examination during one last year and according to the patients’ information. The evaluation of lesions by a dermatologist (=the main investigator of the study) was performed every two months during the study.

### 4.9. The Onset of Atopic Dermatitis

The onset of atopic dermatitis was evaluated according to the patients’ history (the onset of atopic dermatitis under five years of age or later).

### 4.10. The Family History of Atopy

The family history was evaluated according to the patients’ information. We evaluated as positive family history: the occurrence of allergy, atopic dermatitis, asthma bronchiale, rhinoconjunctivitis in parents, brothers, sisters, and children. If there was no family history of these diseases, the family history was evaluated as negative.

## 5. Conclusions

Our study shows the detailed analysis of the sensitization to allergens reagents in patients suffering from atopic dermatitis with the use of a new multiplex assay (ImmunoCAP ALEX2). The high and very high level of specific IgE reached in 75.0% of patients to these 18 molecular components—from PR-10 proteins (Aln g 1, Bet v 1, Cor a1.0103, Cor a1.0401, Fag s 1), lipocalin (Can f 1), NPC2 family (Der f 2, Der p 2), uteroglobin (Fel d 1), from Alternaria alternata (Alt a 1), Beta expansin (Lol p 1, Phl p 1), molecular components from Timothy, cultivated rye (Secc pollen) and peritrophin-like protein domain Der p 23. The high and very high level of specific IgE to other lipocalins such as Fel d 7 and Can f 4, to Art v (mugwort), Bla g 9 (arginine kinase, German cockroach), and to Cyn d (Bermuda grass) reached 52.0% of patients.

The severity of AD is in significant relation to the sensitization to molecular components of storage mites (Gly d 2, Lep d 2—NPC2 family), lipocalins (Can f 1, Can f 2, Can f 4, and Can f 6), arginine kinase (Asp f 6, Bla g 9, Der p 20, Pen m 2), uteroglobin (Fel d 1, Ory c 3), Mn superoxide dismutase (Mala s 11), PR-10 proteins (Fag s 1, Mal d 1, Cor a 1.0401, Cor a 1.0103), molecular components of peritrophin-like domain (Der p 21, Der p 23) and to Secc pollen. In the subgroup of patients suffering from bronchial asthma, the significant role play molecular components from house dust mites and storage mites (Lep d 2, Der p 2, Der f 2—NPC2 family), cysteine protease (Der p 1), peritrophin-like protein domain (Der p 21, Der p 23), Enolase from Alternaria alternata (Alt a 6) and Beta expansin Phl p 1. In patients suffering from rhinitis the important role play Bet v 1 and cross-reactive PR-10 proteins, lipocalin Can f 1, uteroglobin Fel d 1. In patients with the onset of AD under 5 years of age, there is a significantly higher occurrence of sensitization mainly to lipocalins (Can f 1, Can f 2, Can f 4, Cav p 1, Fel d 4, Fel d 7), uteroglobin Fel d 1 and to Asp f 6. The significant relation was confirmed between the persistent eczematic lesions and sensitization to molecular components of storage mites, house dust mite (NPC2 family), Der p 23, Asp f 6, and Phl p 1.

The results of our study demonstrate the detailed profile of sensitization to allergens reagents (allergen extract and molecular components) in patients with atopic dermatitis. We show the significance of disturbed epidermal barrier, resulting in increased penetration of allergens. We confirmed the significant relationship between the severity of AD, the occurrence of bronchial asthma and allergic rhinitis, and high levels of specific IgE to allergen reagents. Our results may be important for regime measures and immunotherapy; Der p 23 shall be considered as an essential component for the diagnosis and specific immunotherapy of house dust mite allergy.

In atopic dermatitis patients, molecular approach is suitable for assessing the risk of potential allergic reactions, which depend on the individual allergic (clinical) sensitization profile, and for evaluating whether unknown potential triggering factors are present. Our results may demonstrate the significance of disturbed epidermal barrier, resulting in increased transepidermal water loss and penetration of allergens, irritants, and microbes.

## Figures and Tables

**Table 1:** The characteristic of patients.

Characteristic of Patients	Number of Patients
Patients suffering from atopic dermatitis	100 patients
Sex	48 men, 52 women
The average age	40.9 years (min. age 14 years, max. age 67 years)
The average SCORAD	39 points, s.d. 13.1 points
Severity of AD	Mild form 14 (14%)
	Moderate form 61 (61%)
	Severe form 25 (25%)
Family history about atopy	Positive family history 48 patients (48%)
	Negative family history 52 patients (52%)
Onset of AD	Under 5 years of age 61 patients (61%)
	After 5 year of age 39 patients (%)
Eczematic lesions	Persistent 57 patients (57%)
	Occasional 43 patients (43%)
Bronchial asthma	55 (55%)
Allergic rhinitis	74 (74%)

**Table 2:** The order of allergen reagents (allergenic extracts and molecular components) according to the frequency of sensitization in 100 atopic dermatitis patients (=100%). We show the sensitization to allergens reagents, which has been recorded in more than 10% of patients.

Allergen Reagents (Allergenic Extracts and Molecular Components)	Sensitization Confirmed in Patients (%)
Phl p 1 (Beta expansin, Timothy grass)	57.0
Bet v 1 (PR-10 protein, birch), Lol p 1 (Beta expansin, rye grass), Sec c_pollen (cultivated rye, pollen)	53.0
Fag s1 (PR-10 protein, European beech)	49.0
Cor a 1.0103 (PR-10 protein, hazel pollen) Cyn d 1 (Beta expansin, Bermuda grass)	48.0
Cor a_pollen (hazel pollen)	47.0
Der f 2 (NPC2 family, house dust mite) Fra a 1 + 3 (PR-10 protein + non-specific lipid transfer protein, type 1, strawberry) Phl p 2 (expansin, Timothy grass)	45.0
Cor a 1.0401 (PR-10 protein, hazel pollen) Cyn d (Bermuda grass) Der p 2 (NPC2 family, house dust mite) Fel d 1 (uteroglobin, cat)	44.0
Aln g 1 (PR-10 protein, alder)	43.0
Pas n (bahia grass) Phl p 5.0101 (grass group 5/6, Timothy grass), Phl p 6 (grass group 5/6, Timothy grass)	42.0
Lep d 2 (NPC2 family, storage mite) Mal d 1 (PR-10 protein, apple)	41.0
Can f 1 (lipocalin, dog) Der p 23 (peritrophin-like protein domain, house dust mite)	36.0
Gly m 4 (PR-10 protein, soybean)	35.0
Der p 1 (cysteine protease, house dust mite)	34.0
Ara h 8 (PR-10 protein, peanut) Gly d 2 (NPC2 family, storage mite)	33.0
Art v (mugwort), Can f 6 (lipocalin, dog)	32.0
Der f 1 (cysteine protease, house dust mite) Fel d 7 (lipocalin, cat)	31.0
Can f_male urine (male dog urine)	30.0
Can f 4 (lipocalin, dog) Equ c 1 (lipocalin, horse)	29.0
Pla l (English plantain)	28.0
Ory c 3 (uteroglobin, rabbit) Phr c (common reed)	27.0
Aca s (storage mite) Alt a 1 (unknown, Alternaria alternata) Der p 5 (unknown, house dust mite) Fel d 4 (lipocalin, cat)	26.0
Amb a (ragweed) Tyr p (storage mite)	25.0
Ach d (house cricket) Der p 7 (mites group 7, bactericidal permeability-increasing-like protein, house dust mite) Loc m (migratory locust) Mala s 11 (Mn superoxide dismutase, Malassezia sympodialis)	24.0
Amb a 4 (plant defensin, ragweed) Der p 20 (arginine kinase, house dust mite), Ten m (mealworm)	23.0 23.0
Api g 1 (PR-10 protein, celery), Mus m 1 (lipocalin and urinary prealbumin, house mouse)	22.0
Bla g 9 (arginine kinase, German cockroach) Can f_Fd1 (uteroglobin, dog), Cav p 1 (lipocalin, guinea pig)	21.0
Asp f 6 (Mn superoxide dismutase, Aspergillus fumigatus) Bla g 4 (calycin, lipocalin, German cockroach) Fra e 1 (Ole e 1-like protein family, European ash), Jug r_pollen (walnut pollen)	20.0
Art v 1 (plant defensin, mugwort) Dau c (carrot) Dau c 1 (PR-10 protein, carrot)	19.0
Par j 2 (non-specific lipid transfer protein, type 1, pellitory of the wall) Pen m 2 (arginine kinase, shrimp) Ves v 5 (antigen 5, yellow jacket venom)	18.0
Ama r (redroot pigweed) Can f 2 (lipocalin, dog) Rat n (rat)	17.0
Api m (honey bee venom) Cup a 1 (pectate lyase, cypress) Fra e (European ash), Gal d_white (egg white) Per a (American cockroach)	16.0
Aca m (acacia) Api m 1 (phospholipase A2, honey bee venom) Asp f 3 (peroxysomal protein, Aspergillus fumigatus) Cla h 8 (mannitol dehydrogenase, cladosporium herbarum) Cry j 1 (pectate lyase, Japanese cedar) Der p 21 (unknown, house dust mite) Hel a (sunflower seed) Hom g (lobster) Pla l 1 (Ole e 1-like protein family, English plantain) Sal k (Russian thistle, saltwort)	15.0
Act d 2 (thaumatin-like protein, kiwi fruit) Mala s 6 (cyclophilin, malassezia sympodialis) Sac c (Saccharomyces cerevisiae) Sol t (potato)	14.0
Api m 10 (icarapin variant 2, honey bee venom) Pha v (green bean, French bean)	13.0
Alt a 6 (enolase, Alternaria alternata) Amb a 1 (pectate lyase, ragweed), Api g 2 (non-specific lipid transfer protein, type 1, celery) Blo t 5 (mites group 5, storage mite) Can s (hemp), Hor v (barley) Lol spp. (squid) Pan b (Northern shrimp), Phl p 7 (polcalcin, Timothy grass) Phod s 1 (lipocalin, Siberian hamster) Sal k 1 (pectin methylesterase, Russian thistle, saltwort) Urt d (nettle)	12.0
Bet v 2 (profilin, birch) Cuc m 2 (profilin, muskmelon) Pec spp. (scallop) Sec c_flour (cultivated rye) Ses i 1 (2S albumin, sesame) Tyr p 2 (NPC2 family, storage mite) Ves v (yellow jacket venom), Ves v 1 (phospholipase A1, yellow jacket venom) Zea m 14 (non-specific lipid transfer protein, type 1, maize)	11.0
Che q (quinoa) Mala s 5 (unknown, Malassezia sympodialis) Ole e 1 (Ole e 1-family, olive) Pers a (avocado) Phl p 12 (profilin, Timothy grass) Pla a 2 (polygalacturonase, London plane tree) Pol d (paper wasp venom) Pyr c (pear) Sola l (tomato) Ulm c (elm) Zea m (maize)	10.0

**Table 3:** Allergen reagents (molecular components) with the level of specific IgE in Classes 3 and 4 with the positivity in 6 and more patients. We also show the sensitization rate of 75 selected molecular components in the classess 0, 1, 2 (analysis according to TURF—total unduplicated reach and frequency).

	The Number of Patients According to the Level of Specific IgE in Classes 0–4 in 75 Selected Allergen Reagents, (100 Patients Included in the Study = 100%)					
Allergen Reagents (Allergen Extract, Molecular Components)	Class 0	Class 1	Class 2	Class 3	Class 4	Class 3 + Class 4
Acas	74	5	7	8	6	14
Achd	76	6	3	7	8	15
Alng1	57	0	12	14	17	31
Alta1	74	0	0	4	22	26
Alta6	88	2	4	4	2	6
Amba	75	5	8	8	4	12
Amba4	77	7	5	8	3	11
Apig1	78	5	3	8	6	14
Apim10	87	6	1	6	0	6
Arah8	67	8	10	8	7	15
Artv	68	5	7	10	10	20
Artv1	81	2	7	9	1	10
Aspf6	80	2	4	9	5	14
Betv1	47	2	6	5	40	45
Blag9	79	0	1	2	18	20
Blot5	88	3	3	3	3	6
Canf_Fd1	79	6	9	6	0	6
Canf_maleurine	70	2	8	7	13	20
Canf1	64	1	8	10	17	27
Canf2	83	1	2	4	10	14
Canf4	71	1	6	5	17	22
Canf6	68	0	6	5	21	26
Cavp1	79	1	4	7	9	16
Cora_pollen	53	4	7	19	17	36
Cora1.0103	52	2	7	7	32	39
Cora1.0401	56	0	14	10	20	30
Cynd	56	12	16	14	2	16
Cynd1	52	8	16	16	8	24
Dauc	81	6	3	7	3	10
Dauc1	81	5	2	6	6	12
Derf1	69	3	5	12	11	23
Derf2	55	1	2	2	40	42
Derp1	66	2	7	7	18	25
Derp2	56	0	2	2	40	42
Derp20	77	2	2	1	18	19
Derp21	85	0	1	2	12	14
Derp23	64	1	7	5	23	28
Derp5	74	8	3	1	14	15
Derp7	76	6	12	3	3	6
Equc1	71	2	8	1	18	19
Fags1	51	3	6	15	25	40
Feld1	56	4	6	2	32	34
Feld4	74	3	8	6	9	15
Feld7	69	5	10	3	13	16
Fraa13	55	6	13	12	14	26
Frae	84	2	4	2	8	10
Frae1	80	4	5	2	9	11
Glyd2	67	9	8	4	12	16
Glym4	65	7	12	8	8	16
Homg	85	2	7	2	4	6
Chispp_	91	1	2	3	3	6
Lepd2	59	4	9	18	10	28
Locm	76	8	6	4	6	10
Lolp1	47	7	6	10	30	40
Malas11	76	0	7	1	16	17
Malas5	90	1	3	1	5	6
Mald1	59	1	17	10	13	23
Musm1	78	1	6	7	8	15
Olee1	90	0	3	2	5	7
Oryc3	73	1	2	10	14	24
Panb	88	0	5	5	2	7
Pasn	58	9	19	12	2	14
Penm2	82	0	6	5	7	12
Phlp1	43	5	9	4	39	43
Phlp2	55	4	9	7	25	32
Phlp5_0101	58	0	5	5	32	37
Phlp6	58	7	6	8	21	29
Phods1	88	3	2	2	5	7
Plal	72	14	2	2	10	12
Plal1	85	3	0	2	10	12
Ratn	83	3	6	6	2	8
Secc_pollen	47	5	17	17	14	31
Tenm	77	7	6	6	4	10
tIgE	18	0	0	0	82	82
Tyrp	75	5	7	8	5	13

**Table 4:** Allergen reagents (molecular components) with the level of specific IgE in Classes 3 and 4 with the positivity in 5 and more patients (according to TURF—total unduplicated reach and frequency). The relation between the level of specific IgE in Classes 3 and 4 and the occurrence of bronchial asthma, allergic rhinitis, the severity of AD, the onset of AD (under 5 years of age or later), family history, and duration of eczematic lesions (persistent or occasional). The levels of significance (*p*-value) are calculated in the chi-square test (at an expected minimum frequency of at least 5) or in Fisher’s exact test. We show only the significant relation (*p*-value < 0.05).

Allergen Reagents, Allergenic Extracts and Molecular Components (Biochemical Name). r = Recombinant, *n*= Natural		Number of Patients	Severity of AD	Bronchial Asthma	Allergic Rhinitis	Onset of AD	Family History	Duration of Lesion
Aca s	Storage mite	14	0.0437	-	-	-	-	0.00316
Ach d	House cricket	15	-	-	-	-	-	0.00164
rAln g 1	PR-10 protein, alder	31	-	-	-	-	-	-
rAlt a 1	Unknown, *Alternaria alternata*	26	-	-	-	-	-	-
rAlt a 6	Enolase, *Alternaria alternata*	6	-	0.0312	-	-	-	-
Amb a	Ragweed	12	-	-	-	-	-	-
rAmb a 4	Plant defensin, ragweed	11	-	-	-	-	-	-
rApi g 1	PR-10 protein, celery	14	-	-	0.0182	-	-	-
rApi m 10	Icarapin variant 2, honey bee venom	6	-	-	-	-	-	
nAra h 1	Cupin (vicillin-type, 7S globulin), peanut	5	0.0349	-	-	-	-	-
nAra h 6	2S albumin, peanut	5	0.0349	-	-	-	-	-
rAra h 8	PR-10 protein, peanut	15	-	-	-	-	-	-
Art v	Mugwort	20	-	-	-	-	-	-
rArt v 1	Plant defensin, mugwort	10	-	-	-	-	-	-
rAsp f 6	Mn superoxide dismutase, *Aspergillus fumigatus*	14	0.00145	-	-	0.00784	-	0.0214
rBet v 1	PR-10 protein, birch	45	-	-	0.009	-	-	-
rBla g 9	Arginine kinase, German cockroach	20	0.00439	-	-	-	-	0.0008
rBlo t 5	Blomia tropicalis, storage mite	6	0.023	-	-	-	-	0.0356
rCan f 1	Lipocalin, dog	27	0.0235	-	0.01	0.00262	-	-
rCan f 2	Lipocalin, dog	14	0.0437	-	-	0.00784	-	-
rCan f 4	Lipocalin, dog	22	-	-	-	0.00623	-	-
rCan f 6	Lipocalin, dog	26	0.00907	-	-	-	-	-
rCanf_Fel d 1-like	Uteroglobin, dog	6	-	-	-	-	-	0.0356
Can f_male urine	Male dog urine	20	-	-	-	-	-	-
rCav p 1	Lipocalin, guinea pig	16	-	-		0.0238		
rCor a 1.0103	PR-10 protein, hazel pollen	39	0.0163	-	0.0163	-	-	0.0482
rCor a 1.0401	PR-10 protein, hazelnut	30	0.00819	-	-	-	-	-
Cor a_pollen	Hazel pollen	36	0.00728	-	0.0164	-	-	0.0211
rCra c 6	Troponin C, North sea shrimp	5	0.0349	-	-	-	-	-
Cyn d	Bermuda grass	16	0.0288	-	-	-	-	-
rCyn d 1	Beta expansin, Bermuda grass	24	0.00168	-	0.0312	-	-	
Dau c	Carrot	10	-	-	-	-	-	0.0402
rDau c 1	PR-10 protein, carrot	12	-	-	0.0329	-	-	-
rDer f 1	Cysteine protease, house dust mite	23	-	-	0.0327	-	-	-
rDer f 2	NPC2 family, house dust mite	42		0.046			0.0497	0.0384
rDer p 1	Cysteine protease, house dust mite	25	-	0.0485	-	-	-	-
rDer p 2	NPC2 family, house dust mite	42		0.046	-	-	0.0497	0.0384
rDer p 20	Arginine kinase, house dust mite	19	0.00224	-	-	-	-	0.00017
rDer p 21	Unknown, house dust mite	14	0.0437	0.0185	-	0.00784	-	0.00316
rDer p 23	Peritrophin-like protein domain, house dust mite	28	0.00785	0.0122	-	-	-	0.00658
rDer p 5	Unknown, house dust mite	15	-	0.0482	-	0.042	-	0.00164
rDer p 7	Bactericidal permeability-increasing-like protein, house dust mite	6	-	0.0312	-	-	-	0.0356
rEqu c 1	Lipocalin, horse	19	-	-	-	-	-	-
rFag s 1	PR-10 protein, European beech	40	0.017	-	0.0189	-	-	0.032
rFel d 1	Uteroglobin, cat	34	0.0319		0.0291	0.00168	0.00117	0.00476
rFel d 4	Lipocalin, cat	15	-	-	-	0.042	0.00713	-
rFel d 7	Lipocalin, cat	16	-	-	-	0.00017	-	-
rFra a 1 + 3	PR-10 protein+ non-specific lipid transfer protein type 1, strawberry	26	-	-	-	-	-	-
Fra e	European ash	10	-	-	-	-	-	-
rFra e 1	Ole e 1-like protein family, European ash	11	-	-	-	-	-	-
rGly d 2	NPC2 family, storage mite	16	0.0455	-	-	-	-	0.00085
rGly m 4	PR-10 protein, soybean	16	-	-	-	-	-	-
Hom g	Lobster	6	0.0337	-	-	-	-	-
Chi spp.	Crab	6	-	-	-	-	-	-
rLep d 2	NPC2 family, storage mite	28	0.0168	0.0462	-	-	-	0.00154
Loc m	Migratory locust	10	-	0.0402	-	-	-	0.0402
nLol p 1	Beta-espansin, Rye grass	40	0.0132		0.0406			0.0106
Lol spp.	Squid	5	-	-	-	-	-	-
rMal d 1	PR-10 protein, apple	23	0.0249	-	-	-	-	-
rMala s 11	Mn superoxide dismutase, Malassezia sympodialis	17	0.00855	-	-	-	-	-
rMala s 5	Unknown, Malassezia sympodialis	6	-	-	-	-	0.0103	-
nMus m 1	Lipocalin and urinary prealbumin, house mouse	15	-	-	-	-	0.00147	-
nOle e 1	Ole e 1-family, olive	7	-	-	-	-	-	0.0185
rOry c 1	Lipocalin, rabbit	5	-	-	-	-	-	-
rOry c 3	Uteroglobin, rabbit	24	0.0431	-	-	-	-	0.041
Pan b	Northern shrimp	7	-	-	-	-	-	-
Pas n	Bahia grass	14	0.0437	-	-	-	-	-
rPen m 2	Arginine kinase, shrimp	12	0.0264	-	-	-	-	0.001
rPer a 7	Tropomyosin, American cockroach	5	-	-	-	-	-	-
rPhl p 1	Beta expansin, Timothy	43	0.0273	0.0298	-	0.0482	-	0.0251
rPhl p 2	Expansin, Timothy	32	0.0164	0.02	-	0.049	-	0.0126
rPhl p 5.0101	Grass group 5/6, Timothy	37	-	-	0.0291	-	-	0.04
rPhl p 6	Grass group 5/6, Timothy	29	-	-	0.0248	-	-	-
rPhod s 1	Lipocalin, Siberian hamster	7	-	-	-	-	-	-
Pla l	English plantain	12	-	-	-	-	-	-
rPla l 1	Ole e 1-like protein family, English plantain	12	-	-	-	-	-	-
Rat n	Rat	8	-	-	-	-	0.0267	-
Sec c_pollen	Cultivated rye, pollen	31	0.0118	0.00971	-	-	-	-
Ten m	Mealworm	10	-	-	-	-	-	0.0402
Tyr p	Storage mite	13	-	0.0338	-	-	-	0.00604
rXip g 1	Beta-parvalbumin, swordfish	5	-	-	-	-	-	-

**Table 5:** The detailed comparison of sensitization to allergen reagents (allergen extracts and molecular component) according to the level of specific IgE (Classes 0, 1, 2, 3, 4) in patients suffering from mild, moderate, and severe form. We show only the significant relation (*p*-value < 0.05).

		Comparison of Column Proportions		
Allegen Reagents	The Level of Specific IgE in Classes 0–4	Mild Form of AD (A)	Moderate Form of AD (B)	Severe Form of AD (C)
Acam	0	.a	C(0.023)	
	1	.a		B(0.022)
Acas	0	C(0.030)	C(0.004)	
Allc	0	.a	C(0.035)	
Alta6	0	.a		
	3	.a		B(0.038)
Amar	0	.a	C(0.016)	
	1	.a		
	2	.a		B(0.010)
Amba	0			
	4	.a		B(0.038)
Apig1	0	C(0.047)	C(0.047)	
Arah1	0	.a	C(0.009)	
	4	.a		B(0.038)
Arah2	0	.a	C(0.038)	
Arah3	0	.a	C(0.038)	
Aspf1	0	.a	C(0.038)	
Aspf6	0	.a	C(0.019)	
	3	.a		B(0.009)
Betv1	0			
	2	B(0.040)		.a
	4			A(0.017)
Betv2	0	.a	C(0.046)	
Blag4	0	.a	C(0.019)	
	1	.a		
	2	.a		B(0.009)
Blag9	0	C(0.025)	C(0.011)	
	2	.a		.a
	3	.a		.a
	4			A(0.025) B(0.001)
Blot21	0	.a	C(0.010)	
Blot5	0		C(0.021)	
Canf1	0	B(0.032) C(0.032)		
Canf2	0	.a		
	3	.a		B(0.038)
Canf6	0	C(0.015)		
	4			A(0.043) B(0.043)
Capa	0	.a	C(0.029)	
Carp	0	.a	C(0.038)	
Clah	0	.a	C(0.002)	
	1	.a		B(0.010)
Clah8	0	.a	C(0.023)	
	4			A(0.015)
Crac6	0		C(0.007)	
Cucm2	0	.a	C(0.046)	
Cupa1	0	.a	C(0.041)	
	1	.a		B(0.035)
Cynd	0	C(0.008)		
Cynd1	0	C(0.016)		
	4	.a		B(0.029)
	1	.a		B(0.035)
Derf2	0	C(0.032)		
Derp20	0	C(0.014)	C(0.007)	
	4			A(0.025) B(0.001)
Derp21	0			
	4	.a		B(0.016)
Derp23	0			
	4	.a		B(0.021)
Dolspp	0	.a	C(0.038)	
Equc1	0	C(0.015)	C(0.021)	
Equc3	0	.a	C(0.038)	
Fags1	0			
	2	B(0.040)		.a
Feld4	0	C(0.008)	C(0.008)	
	4			B(0.008)
Fraa1 + 3	0	C(0.032)		
Frae	0		C(0.011)	
Frae1	0		C(0.016)	
Gadm	0	.a	C(0.038)	
Gald1	0	.a	C(0.002)	
Gald2	0	.a	C(0.001)	
	2	.a		B(0.038)
Gald3	0	.a	C(0.010)	
	2	.a		B(0.038)
Gald5	0	.a	C(0.010)	
Gald_white	0	.a	C(0.008)	
Gald_yolk	0	.a	C(0.002)	
	1	.a		B(0.038)
Glyd2	0			
	4			B(.021)
Glym4	0			
	4	.a		B(.029)
Hela	0	.a	C(.004)	
	1	.a		B(.001)
Hevb8	0	.a	C(.003)	
	4	.a		B(.038)
Horv	0	.a	C(.016)	
Chea	0	.a	C(.002)	
Cheq	0	.a		
	2	.a		B(0.038)
Jugr_pollen	0	.a	C(0.004)	
	1	.a		
	2	.a		B(.002)
Lepd2	0	C(0.017)		
Lolp1	0			
	4			A(0.015) B(0.033)
Lupa	0	.a	C(0.002)	
	2	.a		B(.038)
	3	.a		.a
Mald1	0	C(0.017)		
Malas11	0	.a	C(0.001)	
	4	.a		B(0.008)
Malas6	0	.a	C(0.011)	
	1	.a		B(0.010)
Mani	0	.a	C(0.038)	
Mera1	0	.a	C(0.035)	
Musa	0	.a	C(0.010)	
	1	.a		B(0.035)
Oryc2	0	.a	C(0.035)	
Parj2	0	.a		
	1	.a		
	2	.a		B(0.029)
Pecspp_	0			
	1			
	2	.a		B(0.038)
Penm2	0	C(0.043)	C(0.008)	
Pera	0		C(0.034)	
Pera7	0		C(0.030)	
Persa	0	.a	C(0.002)	
	2	.a		B(0.010)
Phav	0	.a		
	2	.a		B(0.010)
Phlp1	0			
	4			A(0.044) B(0.044)
Phlp12	0	.a	C(0.022)	
Phlp2	0			
	4			B(0.037)
Phlp5_0101	0			
	4			A(0.028)
Phod2	0	.a	C(0.003)	
	3	.a		B(0.038)
Pima	0	.a	C(0.010)	
	1	.a		B(0.035)
Pyrc	0	.a		
	2	.a		B(0.038)
Rajc	0	.a	.a	.a
Rudspp_	0		C(0.030)	
Sacc	0	.a	C(0.011)	
	1	.a		
Salk1	0	.a	C(0.016)	
Scos	0	.a	C(0.035)	
Secc_flour	0	.a	C(0.001)	
	1	.a		B(0.010)
Sesi	0	.a	C(0.035)	
Sesi1	0	.a	C(0.046)	
Solt	0	.a	C(0.011)	
Trifo	0	.a	C(0.035)	
Tyrp	0	C(0.030)	C(0.002)	
Tyrp2	0			
	2			B(0.029)
Urtd	0	.a	C(0.002)	
	1	.a		B(0.001)
Vesv	0	.a	C(0.046)	
	1	.a		B(0.029)
	3	.a		
tIgE	0	B(0.001)		.a
	4		A(0.001)	.a

Comparison of column proportions—Results are based on two-sided tests. For each significant pair, the key of the category with the smaller column proportion appears in the category with the larger column proportion. The significance level for upper case letters (A, B, C): 0.05; .a—this category is not used in comparisons because its column proportion is equal to zero or one.

**Table 6:** Best reach and frequency by group size—18 molecular components with high (Class 3) and very high level of specific IgE (Class 4). **a.** The level of specific IgE in Classes 3 and 4 to these molecular components reached 75 patients (75.0%), the frequency of positivity was at a rate of 632. **b.** The level of specific IgE in Classes 3 and 4 to these molecular components reached 52 patients (52.0%), the frequency of positivity was at a rate of 94. The detailed analysis of this statistic method we show in Figure 1 in the Supplementary Table S2.

Allergen Reagent, Molecular Components	Group Size	Reach	Pct of Cases	Frequency	Pct of Responses
**a**					
Alng1, Alta1, Betv1, Canf1, Cora1.0103, Cora1.0401, Cora_pollen, Derf2, Derp2, Fags1, Feld1, Lolp1, Phlp1, Phlp2, Phlp5_0101, Phlp6, Secc_pollen, Der p 23	18	75	75.0	632	85.8
**b**					
Canf4, Artv, Blag9, Cynd, Feld7	5	52	52.0	94	18

## Data Availability

The data presented in this study are available on request from the corresponding author.

## Supplementary Materials

The following are available online at https://www.mdpi.com/article/10.3390/ijms22105286/s1.
