# Supplementary material for: Analysis of Results of Specific IgE in 100 Atopic Dermatitis Patients with the Use of Multiplex Examination ALEX2—Allergy Explorer
Source: Int J Mol Sci. 2021 May 17;22(10):5286. doi: 10.3390/ijms22105286 (PMC8156228; doi:10.3390/ijms22105286)
Supplement: Supplementary file 1 [file ijms-22-05286-s001.zip › ijms-1114728-supplementary.pdf]

International Journal of
Molecular Sciences

MDPI

Supplementary Materials

Table S1. The detailed overview of 295 allergen reagents (allergenic extracts and molecular components) according to the frequency of sensitisation in classes 0, 1, 2, 3, 4 in 100 atopic dermatitis patients (= 100 %).

**Allergen reagents**	**The number of patients (%) according to the level of specific IgE in classes 0-4 100 patients includde in the study = 100 %**				
	**Class 0**	**Class 1**	**Class 2**	**Class 3**	**Class 4**
Acam	85.0%	10.0%	4.0%	1.0%	0.0%
Acas	74.0%	5.0%	7.0%	8.0%	6.0%
Actd1	91.0%	4.0%	3.0%	2.0%	0.0%
Actd10	96.0%	1.0%	2.0%	0.0%	1.0%
Actd2	86.0%	11.0%	1.0%	2.0%	0.0%
Actd5	99.0%	1.0%	0.0%	0.0%	0.0%
Achd	76.0%	6.0%	3.0%	7.0%	8.0%
Aila	92.0%	5.0%	3.0%	0.0%	0.0%
Allc	94.0%	4.0%	2.0%	0.0%	0.0%
Alls	94.0%	4.0%	2.0%	0.0%	0.0%
Alng1	57.0%	0.0%	12.0%	14.0%	17.0%
Alng4	93.0%	1.0%	4.0%	0.0%	2.0%
Alta1	74.0%	0.0%	0.0%	4.0%	22.0%
Alta6	88.0%	2.0%	4.0%	4.0%	2.0%
Amar	83.0%	8.0%	7.0%	2.0%	0.0%
Amba	75.0%	5.0%	8.0%	8.0%	4.0%
Amba1	88.0%	7.0%	3.0%	0.0%	2.0%
Amba4	77.0%	7.0%	5.0%	8.0%	3.0%
Anao	93.0%	5.0%	1.0%	1.0%	0.0%
Anao2	97.0%	0.0%	3.0%	0.0%	0.0%
Anao3	99.0%	0.0%	0.0%	1.0%	0.0%
Anis1	100.0%	0.0%	0.0%	0.0%	0.0%
Anis3	96.0%	0.0%	0.0%	1.0%	3.0%
Apig1	78.0%	5.0%	3.0%	8.0%	6.0%
Apig2	88.0%	10.0%	0.0%	1.0%	1.0%
Apig6	93.0%	4.0%	2.0%	0.0%	1.0%
Apim	84.0%	8.0%	6.0%	2.0%	0.0%
Apim1	85.0%	11.0%	3.0%	1.0%	0.0%
Apim10	87.0%	6.0%	1.0%	6.0%	0.0%
Arah1	91.0%	1.0%	3.0%	1.0%	4.0%
Arah15	92.0%	4.0%	4.0%	0.0%	0.0%
Arah2	96.0%	1.0%	0.0%	0.0%	3.0%
Arah3	96.0%	0.0%	1.0%	2.0%	1.0%
Arah6	93.0%	1.0%	1.0%	3.0%	2.0%
Arah8	67.0%	8.0%	10.0%	8.0%	7.0%
Arah9	91.0%	3.0%	5.0%	1.0%	0.0%
Argr1	97.0%	0.0%	3.0%	0.0%	0.0%
Artv	68.0%	5.0%	7.0%	10.0%	10.0%
Artv1	81.0%	2.0%	7.0%	9.0%	1.0%
Artv3	92.0%	6.0%	1.0%	0.0%	1.0%
Aspf1	96.0%	2.0%	1.0%	1.0%	0.0%

Aspf3	85.0%	8.0%	5.0%	1.0%	1.0%
Aspf4	97.0%	0.0%	1.0%	1.0%	1.0%
Aspf6	80.0%	2.0%	4.0%	9.0%	5.0%
Aves	93.0%	6.0%	0.0%	1.0%	0.0%
Bere	98.0%	0.0%	2.0%	0.0%	0.0%
Bere1	99.0%	0.0%	1.0%	0.0%	0.0%
Betv1	47.0%	2.0%	6.0%	5.0%	40.0%
Betv2	89.0%	4.0%	4.0%	0.0%	3.0%
Betv6	98.0%	2.0%	0.0%	0.0%	0.0%
Blag1	99.0%	1.0%	0.0%	0.0%	0.0%
Blag2	94.0%	3.0%	1.0%	2.0%	0.0%
Blag4	80.0%	10.0%	9.0%	1.0%	0.0%
Blag5	99.0%	1.0%	0.0%	0.0%	0.0%
Blag9	79.0%	0.0%	1.0%	2.0%	18.0%
Blot10	92.0%	4.0%	0.0%	1.0%	3.0%
Blot21	95.0%	2.0%	3.0%	0.0%	0.0%
Blot5	88.0%	3.0%	3.0%	3.0%	3.0%
Bosd2	93.0%	0.0%	4.0%	3.0%	0.0%
Bosd4	98.0%	1.0%	1.0%	0.0%	0.0%
Bosd5	100.0%	0.0%	0.0%	0.0%	0.0%
Bosd6	97.0%	2.0%	1.0%	0.0%	0.0%
Bosd8	94.0%	3.0%	2.0%	1.0%	0.0%
Bosd_meat	99.0%	1.0%	0.0%	0.0%	0.0%
Bosd_milk	96.0%	2.0%	1.0%	1.0%	0.0%
Bropa	99.0%	1.0%	0.0%	0.0%	0.0%
Camd	98.0%	1.0%	1.0%	0.0%	0.0%
Canf1	64.0%	1.0%	8.0%	10.0%	17.0%
Canf2	83.0%	1.0%	2.0%	4.0%	10.0%
Canf3	93.0%	4.0%	1.0%	1.0%	1.0%
Canf4	71.0%	1.0%	6.0%	5.0%	17.0%
Canf6	68.0%	0.0%	6.0%	5.0%	21.0%
Canf_Fd1	79.0%	6.0%	9.0%	6.0%	0.0%
Canf_maleurine	70.0%	2.0%	8.0%	7.0%	13.0%
Cans	88.0%	3.0%	5.0%	4.0%	0.0%
Cans3	96.0%	0.0%	2.0%	1.0%	1.0%
Capa	92.0%	5.0%	3.0%	0.0%	0.0%
Caph_epithelia	98.0%	1.0%	0.0%	0.0%	1.0%
Caph_milk	97.0%	1.0%	1.0%	0.0%	1.0%
Carc	98.0%	2.0%	0.0%	0.0%	0.0%
Cari	91.0%	1.0%	5.0%	2.0%	1.0%
Carp	96.0%	3.0%	1.0%	0.0%	0.0%
Cavp1	79.0%	1.0%	4.0%	7.0%	9.0%
Cica	95.0%	3.0%	1.0%	1.0%	0.0%
Cits	99.0%	1.0%	0.0%	0.0%	0.0%
Clah	94.0%	5.0%	1.0%	0.0%	0.0%
Clah8	85.0%	14.0%	1.0%	0.0%	0.0%
Cluh	97.0%	0.0%	2.0%	1.0%	0.0%
Cluh1	95.0%	2.0%	0.0%	0.0%	3.0%
Cora1.0103	52.0%	2.0%	7.0%	7.0%	32.0%

Cora1.0401	56.0%	0.0%	14.0%	10.0%	20.0%
Cora11	94.0%	3.0%	3.0%	0.0%	0.0%
Cora14	94.0%	3.0%	2.0%	1.0%	0.0%
Cora8	96.0%	1.0%	2.0%	1.0%	0.0%
Cora9	97.0%	1.0%	2.0%	0.0%	0.0%
Cora_pollen	53.0%	4.0%	7.0%	19.0%	17.0%
Crac6	93.0%	0.0%	2.0%	2.0%	3.0%
Cryj1	85.0%	4.0%	9.0%	1.0%	1.0%
Cucm2	89.0%	2.0%	5.0%	1.0%	3.0%
Cucp	93.0%	4.0%	3.0%	0.0%	0.0%
Cupa1	84.0%	6.0%	8.0%	2.0%	0.0%
Cups	100.0%	0.0%	0.0%	0.0%	0.0%
Cynd	56.0%	12.0%	16.0%	14.0%	2.0%
Cynd1	52.0%	8.0%	16.0%	16.0%	8.0%
Cypc1	94.0%	2.0%	1.0%	0.0%	3.0%
Dauc	81.0%	6.0%	3.0%	7.0%	3.0%
Dauc1	81.0%	5.0%	2.0%	6.0%	6.0%
Derf1	69.0%	3.0%	5.0%	12.0%	11.0%
Derf2	55.0%	1.0%	2.0%	2.0%	40.0%
Derp1	66.0%	2.0%	7.0%	7.0%	18.0%
Derp10	96.0%	0.0%	0.0%	1.0%	3.0%
Derp11	97.0%	1.0%	1.0%	0.0%	1.0%
Derp2	56.0%	0.0%	2.0%	2.0%	40.0%
Derp20	77.0%	2.0%	2.0%	1.0%	18.0%
Derp21	85.0%	0.0%	1.0%	2.0%	12.0%
Derp23	64.0%	1.0%	7.0%	5.0%	23.0%
Derp5	74.0%	8.0%	3.0%	1.0%	14.0%
Derp7	76.0%	6.0%	12.0%	3.0%	3.0%
Dolspp	96.0%	1.0%	3.0%	0.0%	0.0%
Equc1	71.0%	2.0%	8.0%	1.0%	18.0%
Equc3	96.0%	3.0%	1.0%	0.0%	0.0%
Equc4	95.0%	1.0%	2.0%	2.0%	0.0%
Equc_meat	97.0%	3.0%	0.0%	0.0%	0.0%
Equc_milk	97.0%	1.0%	2.0%	0.0%	0.0%
Fage	93.0%	4.0%	3.0%	0.0%	0.0%
Fage2	100.0%	0.0%	0.0%	0.0%	0.0%
Fags1	51.0%	3.0%	6.0%	15.0%	25.0%
Feld1	56.0%	4.0%	6.0%	2.0%	32.0%
Feld2	94.0%	2.0%	4.0%	0.0%	0.0%
Feld4	74.0%	3.0%	8.0%	6.0%	9.0%
Feld7	69.0%	5.0%	10.0%	3.0%	13.0%
Ficb	97.0%	2.0%	0.0%	1.0%	0.0%
Ficc	95.0%	1.0%	4.0%	0.0%	0.0%
Fraa1+3	55.0%	6.0%	13.0%	12.0%	14.0%
Frae	84.0%	2.0%	4.0%	2.0%	8.0%
Frae1	80.0%	4.0%	5.0%	2.0%	9.0%
Gadm	96.0%	1.0%	1.0%	1.0%	1.0%
Gadm1	97.0%	0.0%	1.0%	1.0%	1.0%
Gadm2+3	97.0%	2.0%	1.0%	0.0%	0.0%

Gald1	94.0%	2.0%	3.0%	1.0%	0.0%
Gald2	93.0%	2.0%	4.0%	1.0%	0.0%
Gald3	93.0%	1.0%	4.0%	1.0%	1.0%
Gald4	94.0%	3.0%	1.0%	1.0%	1.0%
Gald5	95.0%	3.0%	1.0%	1.0%	0.0%
Gald_meat	97.0%	2.0%	1.0%	0.0%	0.0%
Gald_white	84.0%	9.0%	3.0%	3.0%	1.0%
Gald_yolk	94.0%	4.0%	1.0%	1.0%	0.0%
Glyd2	67.0%	9.0%	8.0%	4.0%	12.0%
Glym4	65.0%	7.0%	12.0%	8.0%	8.0%
Glym5	99.0%	1.0%	0.0%	0.0%	0.0%
Glym6	97.0%	1.0%	1.0%	1.0%	0.0%
Glym8	100.0%	0.0%	0.0%	0.0%	0.0%
Hela	85.0%	11.0%	3.0%	0.0%	1.0%
Hevb1	99.0%	0.0%	1.0%	0.0%	0.0%
Hevb11	99.0%	1.0%	0.0%	0.0%	0.0%
Hevb3	95.0%	3.0%	1.0%	1.0%	0.0%
Hevb5	98.0%	0.0%	2.0%	0.0%	0.0%
Hevb6_02	95.0%	2.0%	2.0%	1.0%	0.0%
Hevb8	92.0%	2.0%	3.0%	2.0%	1.0%
Homg	85.0%	2.0%	7.0%	2.0%	4.0%
HomsLF	94.0%	2.0%	3.0%	1.0%	0.0%
Horv	88.0%	9.0%	3.0%	0.0%	0.0%
Chea	94.0%	3.0%	3.0%	0.0%	0.0%
Chea1	98.0%	2.0%	0.0%	0.0%	0.0%
Cheq	90.0%	6.0%	4.0%	0.0%	0.0%
Chispp_	91.0%	1.0%	2.0%	3.0%	3.0%
Jugr1	96.0%	1.0%	0.0%	1.0%	2.0%
Jugr2	92.0%	3.0%	4.0%	1.0%	0.0%
Jugr3	96.0%	1.0%	3.0%	0.0%	0.0%
Jugr4	98.0%	0.0%	2.0%	0.0%	0.0%
Jugr6	94.0%	3.0%	3.0%	0.0%	0.0%
Jugr_pollen	80.0%	8.0%	10.0%	1.0%	1.0%
Juna	99.0%	1.0%	0.0%	0.0%	0.0%
Lenc	96.0%	2.0%	1.0%	1.0%	0.0%
Lepd2	59.0%	4.0%	9.0%	18.0%	10.0%
Lits	94.0%	1.0%	2.0%	1.0%	2.0%
Locm	76.0%	8.0%	6.0%	4.0%	6.0%
Lolp1	47.0%	7.0%	6.0%	10.0%	30.0%
Lolspp_	88.0%	4.0%	3.0%	3.0%	2.0%
Lupa	94.0%	2.0%	4.0%	0.0%	0.0%
Maci2SAlbumin	99.0%	0.0%	1.0%	0.0%	0.0%
Macinte	96.0%	3.0%	0.0%	1.0%	0.0%
Mald1	59.0%	1.0%	17.0%	10.0%	13.0%
Mald2	99.0%	0.0%	1.0%	0.0%	0.0%
Mald3	92.0%	0.0%	7.0%	1.0%	0.0%
Malas11	76.0%	0.0%	7.0%	1.0%	16.0%
Malas5	90.0%	1.0%	3.0%	1.0%	5.0%
Malas6	86.0%	5.0%	6.0%	3.0%	0.0%

Mani	96.0%	3.0%	1.0%	0.0%	0.0%
Melg	98.0%	2.0%	0.0%	0.0%	0.0%
Mera1	94.0%	2.0%	1.0%	2.0%	1.0%
Morr	100.0%	0.0%	0.0%	0.0%	0.0%
Musa	93.0%	6.0%	1.0%	0.0%	0.0%
Musm1	78.0%	1.0%	6.0%	7.0%	8.0%
Myte	96.0%	3.0%	0.0%	1.0%	0.0%
Olee1	90.0%	0.0%	3.0%	2.0%	5.0%
Olee9	98.0%	1.0%	0.0%	1.0%	0.0%
Oriv	100.0%	0.0%	0.0%	0.0%	0.0%
Oryc1	92.0%	0.0%	3.0%	4.0%	1.0%
Oryc2	94.0%	1.0%	2.0%	2.0%	1.0%
Oryc3	73.0%	1.0%	2.0%	10.0%	14.0%
Orys	95.0%	5.0%	0.0%	0.0%	0.0%
Ory_meat	97.0%	2.0%	1.0%	0.0%	0.0%
Oste	96.0%	1.0%	1.0%	1.0%	1.0%
Ovia_epithelia	100.0%	0.0%	0.0%	0.0%	0.0%
Ovia_meat	100.0%	0.0%	0.0%	0.0%	0.0%
Ovia_milk	94.0%	3.0%	1.0%	1.0%	1.0%
Panb	88.0%	0.0%	5.0%	5.0%	2.0%
Panm	92.0%	5.0%	3.0%	0.0%	0.0%
Paps	94.0%	1.0%	3.0%	1.0%	1.0%
Paps2SAlbumin	98.0%	0.0%	0.0%	1.0%	1.0%
Parj	98.0%	1.0%	1.0%	0.0%	0.0%
Parj2	82.0%	7.0%	8.0%	2.0%	1.0%
Pasn	58.0%	9.0%	19.0%	12.0%	2.0%
Pecspp_	89.0%	7.0%	4.0%	0.0%	0.0%
Pench	97.0%	3.0%	0.0%	0.0%	0.0%
Penm1	95.0%	0.0%	1.0%	1.0%	3.0%
Penm2	82.0%	0.0%	6.0%	5.0%	7.0%
Penm3	99.0%	0.0%	0.0%	1.0%	0.0%
Penm4	98.0%	0.0%	2.0%	0.0%	0.0%
Pera	84.0%	5.0%	7.0%	4.0%	0.0%
Pera7	92.0%	1.0%	2.0%	2.0%	3.0%
Persa	90.0%	5.0%	5.0%	0.0%	0.0%
Petc	98.0%	2.0%	0.0%	0.0%	0.0%
Phav	87.0%	7.0%	5.0%	1.0%	0.0%
Phlp1	43.0%	5.0%	9.0%	4.0%	39.0%
Phlp12	90.0%	3.0%	4.0%	2.0%	1.0%
Phlp2	55.0%	4.0%	9.0%	7.0%	25.0%
Phlp5_0101	58.0%	0.0%	5.0%	5.0%	32.0%
Phlp6	58.0%	7.0%	6.0%	8.0%	21.0%
Phlp7	88.0%	7.0%	2.0%	1.0%	2.0%
Phod2	92.0%	2.0%	2.0%	4.0%	0.0%
Phods1	88.0%	3.0%	2.0%	2.0%	5.0%
Phrc	73.0%	16.0%	9.0%	2.0%	0.0%
Pima	93.0%	6.0%	1.0%	0.0%	0.0%
Piss	97.0%	1.0%	1.0%	1.0%	0.0%
Pisv1	96.0%	1.0%	3.0%	0.0%	0.0%

Pisv2	99.0%	0.0%	1.0%	0.0%	0.0%
Pisv3	98.0%	2.0%	0.0%	0.0%	0.0%
Plaa1	96.0%	2.0%	2.0%	0.0%	0.0%
Plaa2	90.0%	4.0%	5.0%	1.0%	0.0%
Plaa3	91.0%	5.0%	4.0%	0.0%	0.0%
Plal	72.0%	14.0%	2.0%	2.0%	10.0%
Plal1	85.0%	3.0%	0.0%	2.0%	10.0%
Pold	90.0%	8.0%	1.0%	1.0%	0.0%
Pold5	93.0%	3.0%	2.0%	1.0%	1.0%
Popn	91.0%	7.0%	2.0%	0.0%	0.0%
Pruav	98.0%	1.0%	1.0%	0.0%	0.0%
Prudu	96.0%	4.0%	0.0%	0.0%	0.0%
Prup3	94.0%	1.0%	2.0%	1.0%	2.0%
Pyrc	90.0%	6.0%	4.0%	0.0%	0.0%
Rajc	100.0%	0.0%	0.0%	0.0%	0.0%
RajcParvalbumin	99.0%	0.0%	0.0%	0.0%	1.0%
Ratn	83.0%	3.0%	6.0%	6.0%	2.0%
Rudspp_	92.0%	5.0%	1.0%	1.0%	1.0%
Sacc	86.0%	7.0%	7.0%	0.0%	0.0%
Salk	85.0%	7.0%	7.0%	1.0%	0.0%
Salk1	88.0%	9.0%	3.0%	0.0%	0.0%
Sals	98.0%	0.0%	1.0%	1.0%	0.0%
Sals1	93.0%	2.0%	1.0%	1.0%	3.0%
Scos	94.0%	5.0%	1.0%	0.0%	0.0%
Scos1	94.0%	1.0%	2.0%	0.0%	3.0%
Secc_flour	89.0%	7.0%	3.0%	1.0%	0.0%
Secc_pollen	47.0%	5.0%	17.0%	17.0%	14.0%
Sesi	94.0%	2.0%	1.0%	2.0%	1.0%
Sesi1	89.0%	4.0%	3.0%	1.0%	3.0%
Sin	100.0%	0.0%	0.0%	0.0%	0.0%
Sina1	96.0%	2.0%	2.0%	0.0%	0.0%
Solspp_	96.0%	2.0%	2.0%	0.0%	0.0%
Solt	86.0%	7.0%	3.0%	1.0%	3.0%
Solal	90.0%	6.0%	3.0%	1.0%	0.0%
Solal6	95.0%	1.0%	3.0%	1.0%	0.0%
Susd1	95.0%	1.0%	2.0%	1.0%	1.0%
Susd_epithelia	96.0%	2.0%	1.0%	1.0%	0.0%
Susd_meat	96.0%	1.0%	1.0%	2.0%	0.0%
Tenm	77.0%	7.0%	6.0%	6.0%	4.0%
Thua	95.0%	5.0%	0.0%	0.0%	0.0%
Thua1	93.0%	2.0%	2.0%	0.0%	3.0%
Tria14	99.0%	0.0%	1.0%	0.0%	0.0%
Tria19	100.0%	0.0%	0.0%	0.0%	0.0%
TriaaA_TI	97.0%	1.0%	2.0%	0.0%	0.0%
Trifo	94.0%	3.0%	2.0%	1.0%	0.0%
Tris	100.0%	0.0%	0.0%	0.0%	0.0%
Tyrp	75.0%	5.0%	7.0%	8.0%	5.0%
Tyrp2	89.0%	4.0%	6.0%	1.0%	0.0%
Ulmc	90.0%	5.0%	5.0%	0.0%	0.0%

Urtd	88.0%	7.0%	3.0%	2.0%	0.0%
Vacm	95.0%	4.0%	1.0%	0.0%	0.0%
Vesv	89.0%	8.0%	1.0%	2.0%	0.0%
Vesv1	89.0%	4.0%	5.0%	2.0%	0.0%
Vesv5	82.0%	14.0%	3.0%	0.0%	1.0%
Vitv1	97.0%	0.0%	3.0%	0.0%	0.0%
Xipg1	94.0%	1.0%	0.0%	2.0%	3.0%
Zeam	90.0%	5.0%	4.0%	1.0%	0.0%
Zeam14	89.0%	3.0%	5.0%	3.0%	0.0%
tIgE	18.0%	0.0%	0.0%	0.0%	82.0%

Explanation: Class 0 - negative (< 0.3 kU_A/L); Class 1 – low positivity (0.3-1 kU_A/L); Class 2 – moderate positivity (1-5 kU_A/L); Class 3 – high positivity (5-15 kUA/L); Class 4 – very high positivity (> 15 kU_A/L).

Table S2. The detailed analysis of statistic method. **a**. Best reach and frequency by group size–18 allergen reagents, molecular components. **b.** Best reach and frequency by group size-5 allergen reagents, molecular components.

Variables	Size	Reach	Frequency	Responses
**a**				
ADDED: Betv1	1	45	45	6.1
ADDED: Phlp1<br>KEPT: Betv1	2	58	88	11.9
ADDED: Derp2, Phlp5_0101<br>KEPT: Betv1<br>DROPPED: Phlp1	3	67	124	16.8
ADDED: Feld1<br>KEPT: Betv1, Derp2, Phlp5_0101	4	71	158	21.4
ADDED: Alta1<br>KEPT: Betv1, Derp2, Feld1,<br>Phlp5_0101	5	74	184	25.0
ADDED: Canf1, Derf2<br>KEPT: Alta1, Betv1, Feld1,<br>Phlp5_0101<br>DROPPED: Derp2	6	75	211	28.6
ADDED: Phlp1<br>KEPT: Alta1, Betv1, Canf1, Derf2,<br>Feld1, Phlp5_0101	7	75	254	34.5
ADDED: Derp2<br>KEPT: Alta1, Betv1, Canf1, Derf2,<br>Feld1, Phlp1, Phlp5_0101	8	75	296	40.2
ADDED: Fags1<br>KEPT: Alta1, Betv1, Canf1, Derf2,<br>Derp2, Feld1, Phlp1, Phlp5_0101	9	75	336	45.6
ADDED: Lolp1<br>KEPT: Alta1, Betv1, Canf1, Derf2,<br>Derp2, Fags1, Feld1, Phlp1,<br>Phlp5_0101	10	75	376	51.0
ADDED: Cora1.0103<br>KEPT: Alta1, Betv1, Canf1, Derf2,<br>Derp2, Fags1, Feld1, Lolp1, Phlp1,<br>Phlp5_0101	11	75	415	56.3

ADDED: Cora_pollen<br>KEPT: Alta1, Betv1, Canf1, Cora1.0103, Derf2, Derp2, Fags1, Feld1, Lolp1, Phlp1, Phlp5_0101	12	75	451	61.2
ADDED: Phlp2<br>KEPT: Alta1, Betv1, Canf1, Cora1.0103, Cora_pollen, Derf2, Derp2, Fags1, Feld1, Lolp1, Phlp1, Phlp5_0101	13	75	483	65.5
ADDED: Alng1<br>KEPT: Alta1, Betv1, Canf1, Cora1.0103, Cora_pollen, Derf2, Derp2, Fags1, Feld1, Lolp1, Phlp1, Phlp2, Phlp5_0101	14	75	514	69.7
ADDED: Secc_pollen<br>KEPT: Alng1, Alta1, Betv1, Canf1, Cora1.0103, Cora_pollen, Derf2, Derp2, Fags1, Feld1, Lolp1, Phlp1, Phlp2, Phlp5_0101	15	75	545	73.9
ADDED: Cora1.0401<br>KEPT: Alng1, Alta1, Betv1, Canf1, Cora1.0103, Cora_pollen, Derf2, Derp2, Fags1, Feld1, Lolp1, Phlp1, Phlp2, Phlp5_0101, Secc_pollen	16	75	575	78.0
ADDED: Phlp6<br>KEPT: Alng1, Alta1, Betv1, Canf1, Cora1.0103, Cora1.0401, Cora_pollen, Derf2, Derp2, Fags1, Feld1, Lolp1, Phlp1, Phlp2, Phlp5_0101, Secc_pollen	17	75	604	82.0
ADDED: Derp23<br>KEPT: Alng1, Alta1, Betv1, Canf1, Cora1.0103, Cora1.0401, Cora_pollen, Derf2, Derp2, Fags1, Feld1, Lolp1, Phlp1, Phlp2, Phlp5_0101, Phlp6, Secc_pollen	18	75	632	85.8
**b**				
ADDED: Canf4	1	22	22	4.2
ADDED: Artv, Blag9<br>DROPPED: Canf4	2	36	40	7.7
ADDED: Feld7<br>KEPT: Artv, Blag9	3	43	56	10.7
ADDED: Cynd<br>KEPT: Artv, Blag9, Feld7	4	49	72	13.8
ADDED: Canf4<br>KEPT: Artv, Blag9, Cynd, Feld7	5	52	94	18.0
